# Molecular and Metabolic Regulation of Flavonoid Biosynthesis in Two Varieties of *Dendrobium devonianum*

**DOI:** 10.3390/cimb46120855

**Published:** 2024-12-18

**Authors:** Ran Pu, Yawen Wu, Tian Bai, Yue Li, Xuejiao Li, Nengbo Li, Ying Zhou, Jingli Zhang

**Affiliations:** 1 College of Landscape Architecture and Horticulture, Yunnan Agricultural University, Kunming 650201, China; puran1026@163.com (R.P.); yawenwu@ynau.edu.cn (Y.W.); tbai@ynau.edu.cn (T.B.); 14787798717@163.com (Y.L.); lixuejiao@ynau.edu.cn (X.L.); 2 Institute of Caulis Dendrobii of Longling County, Longling 678300, China; lnb0805@126.com (N.L.)

**Keywords:** *Dendrobium devonianum*, flavonoid biosynthesis, metabolome, transcriptome

## Abstract

*Dendrobium devonianum* is an important medicinal plant, rich in flavonoid, with various pharmacological activities such as stomachic and antioxidant properties. In this study, we integrated metabolome and transcriptome analyses to reveal metabolite and gene expression profiles of *D. devonianum,* both green (GDd) and purple-red (RDd) of semi-annual and annual stems. A total of 244 flavonoid metabolites, mainly flavones and flavonols, were identified and annotated. Cyanidin and delphinidin were the major anthocyanidins, with cyanidin-3-O-(6″-O-p-Coumaroyl) glucoside and delphinidin-3-O-(6″-O-p-coumaroyl) glucoside being the highest relative content in the RDd. Differential metabolites were significantly enriched, mainly in flavonoid biosynthesis, anthocyanin biosynthesis, and flavone and flavonol biosynthesis pathways. Transcriptomic analysis revealed that high expression levels of structural genes for flavonoid and anthocyanin biosynthesis were the main reasons for color changes in *D. devonianum* stems. Based on correlation analysis and weighted gene co-expression network analysis (WGCNA) analysis, *CHS2* (chalcone synthase) and *UGT77B2* (anthocyanidin 3-O-glucosyltransferase) were identified as important candidate genes involved in stem pigmentation. In addition, key transcription factors (TFs), including three bHLH (bHLH3, bHLH4, bHLH5) and two MYB (MYB1, MYB2), which may be involved in the regulation of flavonoid biosynthesis, were identified. This study provides new perspectives on *D. devonianum* efficacy components and the *Dendrobium* flavonoids and stem color regulation.

## 1. Introduction

*Dendrobium devonianum* is an epiphytic herb belonging to the genus *Dendrobium* in the Orchidaceae family. It is one of the medicinal *Dendrobium* species [1] and is commonly known as *Dendrobium* purple-skin due to the distinctive purple coloration of its stem epidermis. This species is mainly distributed in China’s Guangxi, Yunnan, Guizhou Province and southeastern Tibet [2]. As one of the predominant endemic species in southwestern China, its cultivation ranks second only to the widely utilized *Dendrobium officinale* currently available on the market. The chemical composition of *D. devonianum* includes polysaccharides, alkaloids, flavonoids and amino acids, among others, with proven efficacy such as stomach nourishment, antioxidant properties, immune regulation enhancement, liver function improvement and anti-fatigue effects [3,4]. Consequently, it holds great potential for development and possesses significant medicinal value.

Flavonoid are the other major components in *D. devonianum* besides polysaccharides and play an important role in its quality evaluation [5]. Plant flavonoid have a variety of pharmacological activities. The *Dendrobium* spp. plant flavonoid and their glycoside components have been shown to possess antioxidant, anti-tumor, hemostatic, immunomodulatory and other pharmacological effects and are considered to be one of the main pharmacological active substances of *Dendrobium* spp. [6,7]. Flavonoid include a variety of subclasses, such as flavones, flavonols, flavanones, flavanonols, anthocyanidins, flavanones, chalcones, and so on. With advancements in technology, previous studies have investigated the flavonoid content of *Amaranthus gangeticus* [8], *Hippophae rhamnoides* [9], *Gentiana szechenyii* [10], *Hedysari Radix* [11], *Fagopyrum dibotrys* [12], *Dendrobium huoshanense* [13] and other plants. Flavonoid are characterized by their wide variety, complex structure and rich biological activities; hence, further comprehensive research is required to elucidate their complex mechanisms of action and associated signaling pathways [14]. For example, 202 flavonoid compounds have been identified from *Dendrobium officinale,* primarily consisting of flavones and flavonols [15]. A total of 364 flavonoid metabolites have been identified from the roots, stems, leaves and flowers of *Hemerocallis citrina*, with significant differences in flavonoid metabolism levels among these parts [16]. Similarly, 101 flavonoid metabolites were identified in the fruits, leaves, flowers and seeds of *Ziziphus jujuba* and *Ziziphus spinosa*, including flavones, flavonols, flavanones, isoflavones, flavanonols, chalcone, flavonols, flavonoid glycosides and phenolic acids [17]. Anthocyanidins are widely present in plants and participate in color formation [18]. Common anthocyanidins include cyanidin chloride, pelargonidin chloride, delphinidin chloride, peonidin chloride, petunidin chloride and Malvidin chloride, which can make plants appear in various colors such as red, purple and blue [19,20]. The brilliant and colorful colors of ornamental plants mainly depend on the formation and accumulation of anthocyanidins, flavones, flavonol metabolites. However, there are currently no reports on the analysis or identification of flavonoids of *D. devonianum* using metabolomics technology.

The formation and accumulation of flavonoid metabolites in plants are closely linked to the related biosynthesis pathway [21], which can be divided into the phenylpropanoid biosynthesis, flavonoid biosynthesis, anthocyanin biosynthesis, and flavone and flavonol biosynthesis pathway according to the product formation process [22]. Key genes involved in these pathways have been identified, such as phenylalanine ammonia-lyase, chalcone synthase, chalcone isomerase, flavanone 4-reductase, naringenin 3-dioxygenase, flavonoid 3′,5′-hydroxylase, flavonoid glucosyltransferase, anthocyanidin synthase. These structural genes play a major role in regulation flavonoid biosynthesis [23,24]. Several transcription factors are known to regulate flavonoid biosynthesis, mainly MYB, bHLH and WD40 [25,26]. Studies based on multi-omics approaches to analyze the synthesis and regulatory mechanisms of flavonoid metabolites have been widely reported. For example, Zheng Tao integrated metabolomics and transcriptomics analyses to explain the metabolic pathways and candidate genes involved in flavonoid biosynthesis of *Zanthoxylum bungeanum* [27]. Qiu Yujie investigated transcriptomics and flavonoid targeted metabolomics were used to study the genes and metabolites of purple and white flowers at different flowering stages of *Dendrobium nobile*, and mined the related pathways and potential functional genes that may lead to the changes in flavonoids and flower colors [28]. In apricot fruits, metabolomics and transcriptomics analysis revealed that apricot fruit flavonoid may be regulated by 17 coding genes and found that three TFs were highly correlated with two structural genes [29].

Much research has also been reported on the dynamic changes in plant colors. In *Toona sinensis*, structural genes such as *DFR*, *ANS* and *UFGT1* may be directly or indirectly regulated by transcription factors SOC1 (MADS-box), CPC (MYB) and bHLH162 (bHLH) to regulate anthocyanin accumulation [30]. The differentially expressed genes in white to purple region of *Centaurea cyanus* were enriched in the phenylpropane biosynthesis pathway and flavonoid biosynthesis pathway, and it revealed that CcMYB6-1 up-regulates the promoter activity of flavonoid 3-hydroxylase (*CcF3H*) and flavanone 4-reductase (*CcDFR*) and collaborates with CcbHLH1 to regulate anthocyanin biosynthesis [31]. Multiple transcription factors such as MYB73, MYB61, bHLH14 and bHLH106 were identified in *Paphiopedilum hirsutissimum*, which may be the key structural genes regulating the formation of flower color [32]. In the study of potato tuber pulp, the expression of the flavonoid gene *StDFR* was found to be enhanced in colored potato pulp [33]. These studies have greatly advanced our understanding of the underlying molecular mechanisms of color change in plants. In this study, the *D. devonianum* purple-red stems were discovered for the first time, but the underlying molecular regulatory mechanisms behind this color variation, as compared to the normal green stems, remain unknown.

In this study, we integrated metabolomics and transcriptomics to analyze metabolite profiles of both green and purple-red stems of *D. devonianum*, with a focus on mining the differences in flavonoid synthesis and key regulatory genes. Our results preliminarily elucidate the molecular regulatory mechanism of the flavonoid biosynthesis pathway in *D. devonianum*. This study not only aids in the cultivation of *Dendrobium* varieties with higher flavonoid content but also offers new insights for the development of pharmacologically active components of *D. devonianum*.

## 2. Materials and Methods

### 2.1. Plant Materials and Treatments

**Figure 1:**
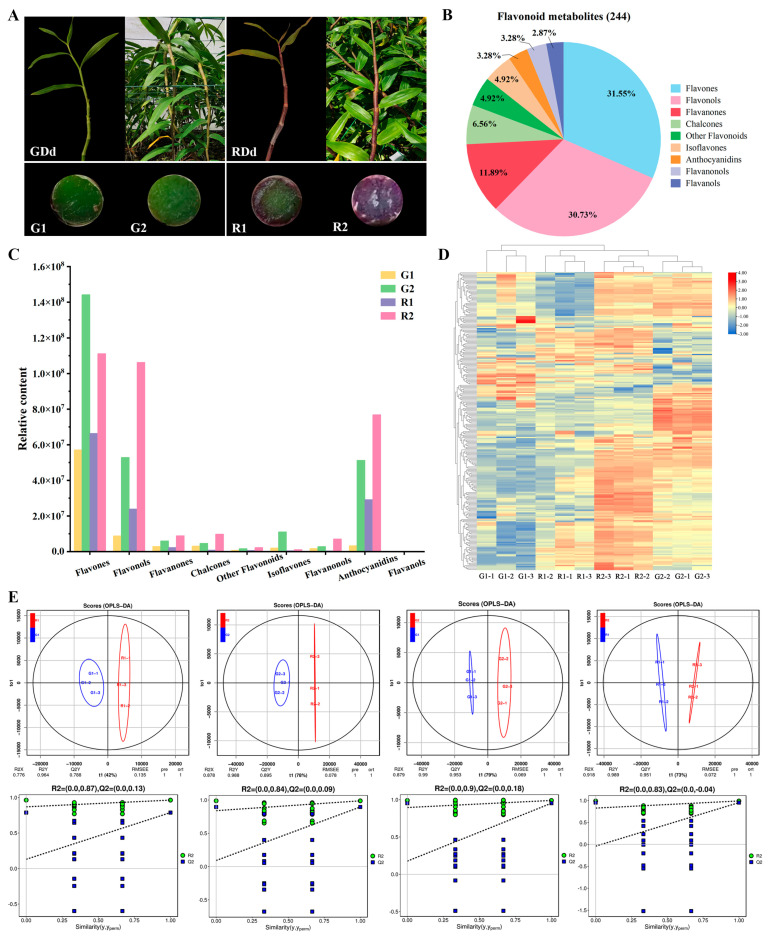
Plant material and identification of flavonoid metabolites. (**A**) Experimental material: *D. devonianum* with green stems (GDd) and purple-red stems (RDd). (G1 and R1 are half-yearly growth period, G2 and R2 are one-yearly growth period). (**B**) Classification statistics of identified flavonoid metabolites. (**C**) Relative content of flavonoid metabolites in 4 samples. (**D**) Clustering heat map of 244 flavonoid metabolites. (**E**) Plot of OPLS-DA scores for each comparison group and point plots for validation of OPLS-DA using the 500 response method.

In this experiment, two varieties of *D. devonianum* were used as test material, namely green *D. dev onianum* (GDd) and purple-red *D. devonianum* (RDd) stems, which were both provided by the Institute of Caulis Dendrobii of Longling County, Longling County, Baoshan City, Yunnan Province, China. Two distinct developmental stages, semi-annual (G1/R1) and annual (G2/R2), were used for metabolic and transcriptome analysis. Stem tissues were collected from three independent plants (three biological replicates), and all samples were immediately frozen in liquid nitrogen and stored at −80 °C (Figure 1A).

### 2.2. Extraction Identification, Analysis of Metabolites and Screening of Differentially Accumulated Metabolites

The freeze-dried samples were crushed with a mixer mill for 1 min at 60 Hz. Then, a precise amount of each individual sample was weighed and transferred to an Eppendorf tube. Extract solution (methanol/water = 3:1, precooled at −40 °C, containing internal standard) was added, and the mixture was extracted overnight at 4 °C on a shaker. Then, it was centrifuged at 12,000 rpm for 15 min at 4 °C. The supernatant was carefully filtered through a 0.22 microporous membrane (SCAA-104, 0.22 μm pore size; ANPEL, Shanghai, China, https://www.anpel.com.cn/ (accessed on 8 June 2023) before LC–MS/MS analysis). The quality control (QC) sample was prepared by mixing an equal aliquot of the supernatants from all of the samples. The UHPLC separation was carried out using an EXIONLC System (Sciex). An amount of 2 μl of samples was injected onto a Waters ACQUITY UPLC HSS T3 C18 column (2.1 × 100 mm, 1.8 µm) operating at 40 °C and a flow rate of 0.4 mL/min. The mobile phase used was acidified water (0.04% acetic acid) (Phase A) and acidified acetonitrile (0.04% acetic acid) (Phase B). Compounds were separated using the following gradient: 95:5 Phase A/Phase B at 0 min; 5:95 Phase A/Phase B at 11.0 min; 5:95 Phase A/Phase B at 12.0 min; 95:5 Phase A/Phase B at 12.1 min; 95:5 Phase A/Phase B at 15.0 min. The effluent was connected to an ESI-triple quadrupole-linear ion trap (Q TRAP)–MS.

LIT and triple quadrupole (QQQ) scans were acquired on a triple quadrupole-linear ion trap mass spectrometer (Q TRAP), AB Sciex QTRAP6500 System, equipped with an ESI-Turbo Ion Spray interface, operating in a positive ion mode and controlled by Analyst 1.6.1 software (AB Sciex). The operation parameters were as follows: ESI source temperature 500 °C; ion spray voltage (IS) 5500 V; curtain gas (CUR) 25 psi; the collision activated dissociation (CAD) was set highest. QQQ scans were acquired as MRM experiments with optimized declustering potential (DP) and collision energy (CE) for each individual MRM transition. The m/z range was set between 50 and 1000. The mass spectrometry data were analyzed for peak identification and extraction, etc. Analyst 1.6.1 was used for the UPLC-MS data filtering, peak detection, alignment and calculations.

Furthermore, a principal component analysis (PCA) was applied to all samples using R package models at http://www.r-project.org/ (accessed on 10 August 2023). Orthogonal partial least squares discriminant analysis (OPLS-DA) was applied in comparison groups using R package models at http://www.rproject.org/ (accessed on 10 August 2023). Differential accumulation of flavonoid metabolites (DAFs) was screened under the following conditions: Variable importance in projection (VIP) value ≥ 1 and a fold-change ≥ 2 or *p*-value ≤ 0.05.

### 2.3. RNA Extraction, Transcriptome Sequencing and Denovo Assembly

Total RNA was extracted using a Trizol reagent kit (Invitrogen, Carlsbad, CA, USA) from *D. devonianum* stem. RNA quality was assessed using an Agilent 2100 Bioanalyzer (Agilent Technologies, Palo Alto, CA, USA) and checked using RNase-free agarose gel electrophoresis. Three stems samples from each sample were used to construct 12 *D. devonianum* transcriptome libraries, which were sequenced using Illumina novaseq 6000 by Gene Denovo Biotechnology Co. (Guangzhou, China). Reads obtained from the sequencing machines included raw reads containing adapters or low quality. Quality control of the raw data was performed, and reads were further filtered to obtain high-quality clean data using fastp (version 0.18.0) [34]. Raw sequencing reads were filtered by removing reads containing aptamers or more than 10% unknown nucleotides (N) and low-quality reads [i.e., more than 50% low-quality (Q < 20) bases]. Transcripts were obtained by de novo assembly of the unaided transcriptome using Trinity software version 2.8.6 for clean data [35].

### 2.4. Transcriptome Data Analysis

Gene expression levels were calculated on the basis of the fragments per kilobase of transcript per million fragments mapped (FPKM) value. Principal component analysis (PCA) was performed with R package models at http://www.r-project.org/ (accessed on 15 August 2023) in this experience. Analysis of differentially expressed genes (DEGs) RNAs differential expression analysis was performed by DESeq2 software (1.20.0) [36] between two different groups and by edgeR [37] between two samples (G1 vs. R1, G2 vs. R2, G1 vs. R1, G2 vs. R2). Genes with |log2 (fold-change)| ≥ 1 and FDR < 0.05 were screened as DEGs, which were subsequently functionally annotated via Gene Ontology (GO) and Kyoto Encyclopedia of Genes and Genomes (KEGG) pathway analyses.

### 2.5. Weighted Gene Co-Expression Network Analysis (WGCNA)

The WGCNA package in R language was used to construct a co-expression network of genes, and all screened differentially accumulated metabolites and DEGs were imported into WGCNA. All genes were divided into different modules according to the expression pattern, and the genes in the same module were absolutely related. The module eigengene value of each module is the first principal component of the module and can represent the gene expression pattern in the module. The correlation and significance of gene matrix values and differentially accumulated metabolites were calculated by the Significance function inside the R language WGCNA package to determine the core modules of differentially accumulated metabolites, and core genes of the core module were analyzed with the differentially accumulated metabolites using the correlation network tool. The results obtained from the analysis were imported into Cytoscape software version 3.10.3 for visual display.

### 2.6. Quantitative Real Time-PCR Validation

Eight key genes in the KEGG pathway were selected for RT-qPCR to validate the reliability of the transcriptome data. The actin gene of *D. devonianum* was selected as an internal reference control, and three technical replicates of each gene were performed each time, totaling three biological replicates. The primer sequences (Supplementary Table S1) were added, and PCR amplification was performed. The relative expression levels of the genes were determined using the cyclic threshold method 2^−∆∆Ct^. The cDNA was used as a template for gene expression determination based on the primer sequences of each gene. RNA was extracted from *D. devonianum* stems using a plant polysaccharide polyphenol RNA extraction kit, and first-strand cDNA was obtained using a cDNA reverse transcription kit.

### 2.7. Statistical Analysis

Statistical analyses were performed using SPSS Statistics 25.0, and Pearson correlation coefficients between differential metabolites and DEGs were calculated using correlation analysis. In addition, clustered heatmaps were plotted using TBtools, and histograms were plotted using Origin2021.

## 3. Results

### 3.1. Metabolome Profiling of D. devonianum Stems

Based on UPLC-MS broad-targeted metabolomics for the detection of metabolites in annual green and purple-red stems of D. devonianum, a total of 974 metabolites were detected, including 196 amino acids and derivatives (20.12%), 124 flavonoids (12.73%), 99 organic acids and derivatives (10.16%), 93 carbohydrates and its derivatives (9.55%), 86 lipids (8.83%), 72 organoheterocyclic compounds (7.39%), 63 nucleotide and its derivates (6.47%), 45 terpenoids (4.62%), 35 phenols and its derivatives (3.59%), 32 phenolic acids (3.29%) and 129 other metabolites (13.25%) (Supplementary Figure S1A). There were significant differences in metabolites, among which flavonoid, organic acids and derivatives, carbohydrates and their derivatives, and nucleotide and their derivates were abundant in the purple-red stems. Amino acids and derivatives, lipids, organoheterocyclic compounds, terpenoids, and phenols and their derivatives were abundant in green stems.

To better reveal the differences in flavonoid metabolites between GDd and RDd at different growth stages, flavonoid targeted metabolomic analyses were performed on G1, G2, R1 and R2 stems. A total of 244 flavonoid metabolites were identified and annotated (Supplementary Table S2), encompassing 77 flavones (31.55%), 75 flavonols (30.73%), 29 flavanones (11.89%), 16 chalcones (6.56%), 12 isoflavones (4.92%), 8 anthocyanidins (3.28%), 8 flavanonols (3.28%), 7 flavonols (2.87%) and 12 other flavonoids (4.92%) (Figure 1B). Among the flavonoid metabolites detected, flavones and flavonols such as luteolin, apigenin, tricin, quercetin, kaempferol, isorhamnetin, and their derivatives were abundant in D. devonianum stems. The flavone content accounts for the largest proportion of the total flavonoid metabolites, and the relative content is highest in G2. The relative content of flavonols, flavanones, chalcones, flavanonols and anthocyanidins was higher in R2 (Figure 1C). Anthocyanidins usually exist in the form of anthocyanins. In this study, a total of eight anthocyanidins were identified in D. devonianum stems, mainly cyanidin and delphinidin glycosides. Among them, the relative content of cyanidin-3,5-O-diglucoside, delphinidin-3,5,3′-Tri-O-glucoside, delphinidin-3-O-rutinoside-7-O-glucoside and cyanidin-3-O-(6″-O-sinapoyl) sophoroside-5-O-glucoside was higher in RDd and showed a clear accumulation trend. The relative content of cyanidin-3-O-(6″-O-p-Coumaroyl)glucoside in R2 is relatively high. Delphinidin-3-O-(6″-O-p-coumaroyl)glucoside and cyanidin-3-O-(6″-O-malonyl) glucoside-5-O-glucoside accumulated in both GDd and RDd, but the relative content in RDd was higher than that in GDd (Supplementary Figure S1B). Overall, the content of anthocyanins in green stems is relatively low.

Additionally, the cluster heat map analysis based on normalized accumulation of 244 flavonoid metabolites showed that the sample groups had good reproducibility, 12 samples were clearly separated into four clusters, and the relative contents of total flavonoid at different periods were significantly different in GDd and RDd (Figure 1D). Principal component analysis (PCA) results exhibited that the samples were clearly separated from each other, indicating that flavonoid were accumulated and expressed in different patterns in the two growth periods of GDd and RDd, and the different replicates showed strong correlation, suggesting that the samples had good replications (Supplementary Figure S1C). To further clarify the differences in flavonoid metabolites among different samples of *D. devonianum*, the metabolome data were analyzed, and scores were plotted according to the Orthogonal Partial Least Squares Discriminant Analysis (OPLS-DA) model. The results showed that the OPLS-DA model had good discriminative ability without overfitting (Supplementary Table S3), and the grouping of the four comparative groups was feasible (Figure 1E).

### 3.2. Screening and Identification of Differential Metabolites

**Figure 2:**
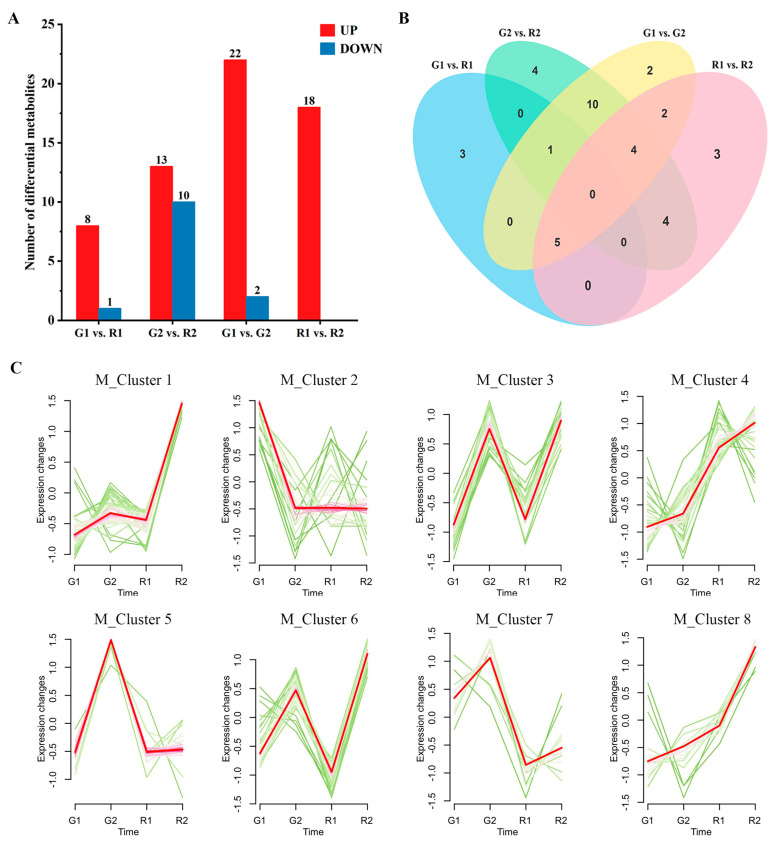
Identification of differential metabolites: (**A**) Identification of the number of up-regulated (UP) and down-regulated (DOWN) differential metabolites (DAFs) among the four comparative groups. (**B**) Venn diagram of differential metabolites identified in comparison groups (G1 vs. R1, G2 vs. R2, G1 vs. G2 and R1 vs. R2). (**C**) Analysis of flavonoid metabolite expression module.

Differential accumulation of flavonoid metabolites (DAFs) was screened for four comparison groups (G1 vs. R1, G2 vs. R2, G1 vs. G2, R1 vs. R2), using a fold-change of ≥2 and a VIP of ≥1 as conditions. A total of 38 DAFs were screened (Supplementary Table S4). Among them, most DAFs come from G1 vs. G2; a total of 24 were identified, of which 22 were up-regulated and 2 were down-regulated. This was followed by 23 from G2 vs. R2, of which 13 were up-regulated and 10 were down-regulated. The lowest was from G1 vs. R1, with only nine, including eight up-regulated and one down-regulated. Only 18 were up-regulated in R1 vs. R2 (Figure 2A). The number of up-regulated metabolites was higher than that of down-regulated metabolites in all four comparison groups, indicating that more metabolites were present in the annual stems and red stems.

KEGG enrichment analysis of DAFs revealed that they were mainly enriched in the biosynthesis of secondary metabolites, flavonoid biosynthesis, flavone and flavonol biosynthesis, and anthocyanin biosynthesis pathway (Supplementary Figure S2). Meanwhile, we screened 16 significantly different metabolites annotated on these four pathways, which may play an important role in stem color difference phenotypes (Supplementary Table S5). Venn diagram analysis found that G2 vs. R2 contained four unique DAFs, namely quercetin-3-O-(4″-O-glucosyl) rhamnoside, quercetin-3-O-rutinoside, isoschaftoside and apigenin-6-C-(2″-glucosyl) arabinoside (Figure 2B). Statistical analysis of the DAFs showed a gradual increase in the type and content of metabolites as the *D. devonianum* matured. The significant differential metabolites in G2 vs. R2 and G1 vs. G2 were mainly flavones, suggesting that the flavone compounds were accumulated in large quantities in the annual *D. devonianum* stems. There was no accumulation of anthocyanidins in G1 vs. R1, and with the maturation of the stems, anthocyanidins continued to accumulate, which made the plants a purplish-red color. In addition, the significant differential metabolites in G1 vs. R1 and R1 vs. R2 were mainly flavonols, and flavonol compounds accumulated more in the RDd, and the deepening of the color as the RDd matured might be caused by flavonol compounds (Supplementary Figure S3).

In order to deeply analyze the accumulation characteristics of flavonoid metabolites in *D. devonianum*, we performed a trend clustering analysis. The results showed that the 244 flavonoid metabolites could be categorized into eight enrichment patterns (Figure 2C). We noticed that a large number of metabolites with high accumulation in R2 were mainly enriched in M_Cluster 1 and M_Cluster 8, which were mainly dominated by flavones and flavonols. Among them, kaempferol-3-O-robinobioside, kaempferol-3-O-rutinoside, cyanidin-3-O-(6″-O-p-Coumaroyl) glucoside and luteolin-7-O-rutinoside were accumulated in large quantities in R2. Delphinidin-3,5,3′-Tri-O-glucoside, quercetin-3-O-rutinoside and cyanidin-3,5-O-diglucoside were significantly up-regulated during the growth of RDd. In contrast, metabolites highly accumulated in G2 were enriched in M_Cluster 5 and M_Cluster 7, which were mainly flavones. Among them, apigenin-8-C-glucoside, apigenin-6-C-glucoside and apigenin-7-O-rutinoside were accumulated in large quantities in G2. Based on the above analysis, we speculate that DAFs in the biosynthetic pathways of flavonoid metabolites may cause changes in stem color during the development of *D. devonianum*.

### 3.3. Transcriptome Sequencing, Assembly and Statistics

To investigate the molecular regulation mechanism of flavonoid biosynthesis in GDd and RDd, transcriptome sequencing analysis was performed on 12 samples. By high-throughput sequencing, 36,835,456~57,266,416 raw sequences (Raw reads) were obtained for each sample. After filtering low-quality reads, 36,730,894~57,030,448 sequences (Clean reads) were obtained for each sample. After further splicing the high-quality clean reads in the 12 samples, the final number of Unigenes was 127,024, with an average length of 687 bp and an N50 length of 1262 bp. The lowest values of Q20 and Q30 were 96.82% and 91.34%, respectively, and the average GC content was 44.7%, indicating that the sequencing data were of high quality and reliable and could be used for further studies (Supplementary Table S6). Meanwhile, the PCA analysis of 12 samples (Supplementary Figure S4A) and the results of the correlated heat map (Supplementary Figure S4B) once again supported the reliability of our transcription data.

### 3.4. Identification and Functional Enrichment Analysis of the DEGs

**Figure 3:**
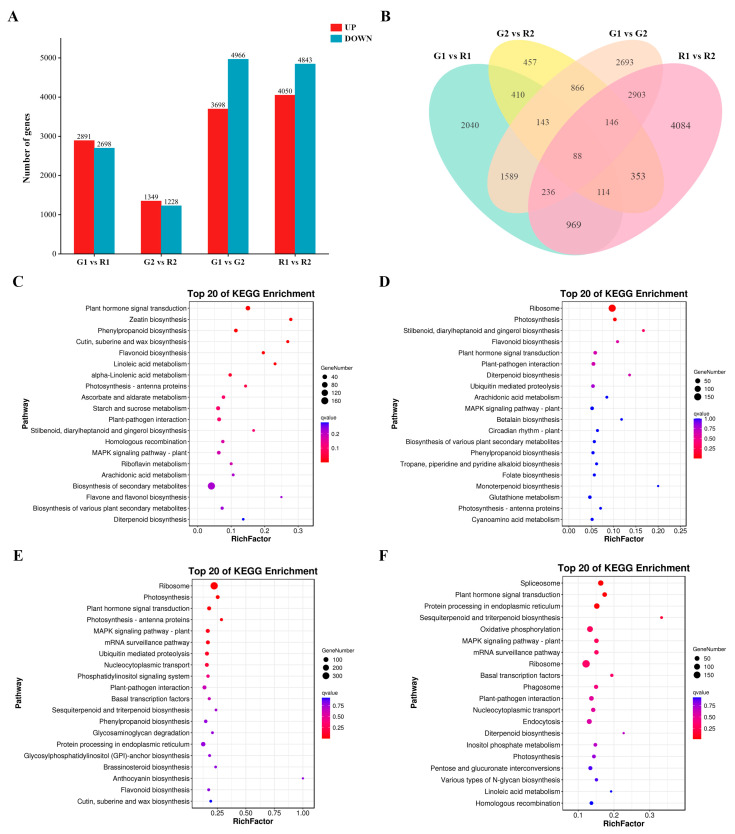
Analysis of DEGs: (**A**) Statistics of the number of up-regulated (UP) and down-regulated (DOWN) DEGs among the four comparative groups. (**B**) Venn diagrams of DEGs in four comparison groups (G1 vs. R1, G2 vs. R2, G1 vs. G2 and R1 vs. R2). (**C**) KEGG enrichment analyses of DEGs in the G1 vs. R1. (**D**) KEGG enrichment analyses of DEGs in the G2 vs. R2. (**E**) KEGG enrichment analyses of DEGs in the G1 vs. G2. (**F**) KEGG enrichment analyses of DEGs in the R1 vs. R2.

Transcriptome data showed that drastic gene expression changes occurred during the growth and development of *D. devonianum*. Differential expression genes (DEGs) were screened with |log2 (fold-change)| > 1 and FDR < 0.05. There were 5589, 2577, 8664 and 8893 DEGs between the G1 vs. R1, G2 vs. R2, G1 vs. G2 and R1 vs. R2 comparison groups, respectively. There were 11,988 and 13,735 DEGs, of which 2891, 1349, 3698 and 4050 were up-regulated DEGs, and 2698, 1228, 4966 and 4843 were down-regulated DEGs, respectively (Figure 3A). Venn diagram analysis found that 88 DEGs (Supplementary Table S7) in the four comparison groups were continuously differentially expressed during the growth of GDd and RDd (Figure 3B). These included *UGT73E1* (UDP-glycosyltransferase73E1; GenBank: AY345979.1), which showed significant up-regulation in GDd but significant down-regulation in RDd. *GALS1* (galactose β-14-galactosyltransferase; GenBank. KJ13885.l), associated with rhamnose synthesis, was significantly down-regulated in GDd but up-regulated in RDd during maturation. We hypothesize that these consistently differentially expressed DEGs may play an important role in stem color formation in *D. devonianum*.

In order to further understand the differential genes involved in flavonoid biosynthesis, DEGs were further assigned to the KEGG pathway. The KEGG enrichment analysis revealed that these DEGs significantly enriched in several pathways including biosynthesis of secondary metabolites (ko01110), ribosome (ko03010), carbon metabolism (ko01200), phenylpropanoid biosynthesis (ko00940), flavonoid biosynthesis (ko00941), flavone and flavonol biosynthesis (ko00944), and anthocyanin biosynthesis pathway (ko00942) (Figure 3C–F). We selected four enriched pathways related to the biosynthesis of flavonoid, namely the ko00940, ko00941, ko00942 and ko00944. The four comparison groups enriched 60, 28, 1 and 6 DEGs in these four pathways, respectively.

### 3.5. Gene Expression Trend Analysis

**Figure 4:**
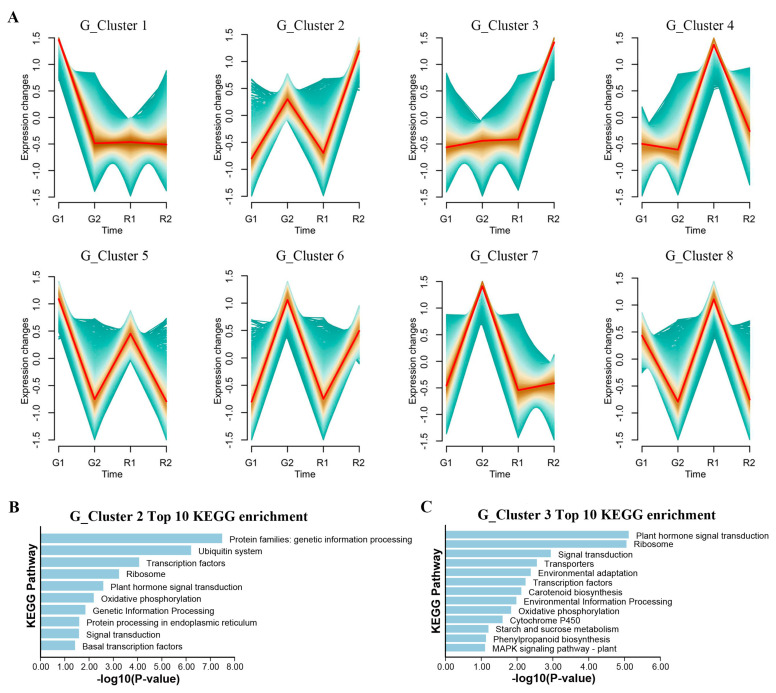
Gene trend expression analysis: (**A**) Analysis of genes clustering trend. (**B**) G_Cluster 2 KEGG enrichment analysis. (**C**) G_Cluster 3 KEGG enrichment analysis.

In order to understand the changing rules of genes during the maturation of *D. devonianum*, a trend clustering analysis of gene expression abundance at different growth stages was conducted. Genes were clustered into eight expression patterns (Figure 4A). Notably, the expression trends of G_Cluster 2 (2692 genes) and G_Cluster 3 (3344 genes) showed highly similar to the accumulation trends of flavonoid metabolites M_Cluster 1 and M_Cluster 6, which implied a synergy between the regulation of gene expression and metabolite accumulation. G_Cluster 2 increased in maturity and showed a decreasing trend in immaturity, and G_Cluster 3 showed no significant change in GDd and significant up-regulation in RDd. KEGG enrichment analysis of genes in G_Cluster 2 and G_Cluster 3 showed that DEGs in G_Cluster 2 were mainly enriched in the pathways of the protein families’ genetic information processing, ubiquitin system, transcription factors, ribosome and plant hormone signal transduction (Figure 4B). DEGs in G_Cluster 3 were mainly enriched in Plant hormone signal transduction, ribosome, signal transduction, transporters and environmental adaptation (Figure 4C). These results suggest that the accumulation of flavonoid metabolites may be accompanied by regulation of plant hormone levels and transcription factors, indirectly contributing to the differences between green and red stems.

### 3.6. Identification of the Key Genes for Flavonoid Biosynthesis in D. devonianum

**Figure 5:**
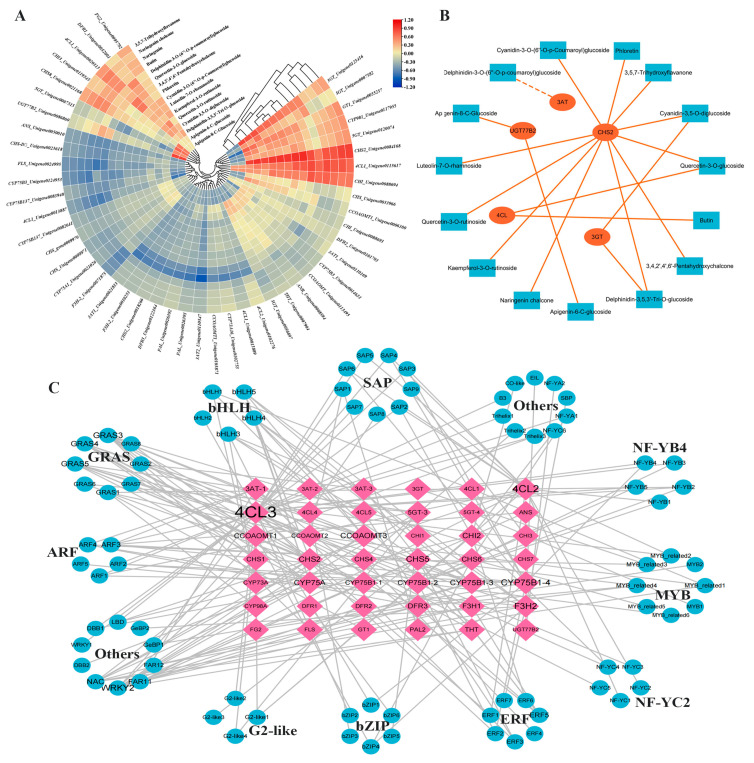
(**A**) Correlation analysis of flavonoid metabolites with flavonoid biosynthesis genes (red color indicates high correlation, blue color indicates low correlation). (**B**) Flavonoid structural gene and metabolites network interactions graph (blue boxes indicate metabolites, orange circles indicate gene). (**C**) Network diagram of the correlation between key structural gene and TFs (pink diamonds indicate DEGs, blue circles indicate TFs).

To investigate the key genes involved in the biosynthesis of flavonoid metabolites in *D. devonianum* stems, based on the database and basic functional annotation information, we screened and identified relevant genes in the phenylpropanoid biosynthesis, flavonoid biosynthesis, anthocyanin biosynthesis, and flavone and flavonol biosynthesis pathway. A total of 47 related genes were identified, regulating 21 enzymes, namely *PAL*, *4CL*, *CHS*, *CYP73A*, *CYP98A*, *CYP75B1*, *CYP75A*, *CHI*, *F3H*, *DFR*, *CCOAOMT*, *ANS*, *ANR*, *FLS*, *THT*, *UGT77B2*, *3GT*, *5GT*, *3AT*, *GT1*, *FG2* (Supplementary Table S8). We correlated 47 genes with 16 flavonoid metabolites that significantly accumulated in *D. devonianum* (Figure 5A) and plotted network interactions (R ≥ |0.8|) (Figure 5B). The results showed that five genes were significantly correlated with 15 flavonoid metabolites, including *CHS2* with 3,4,2′,4′,6′-pentahydroxychalcone, phloretin, cyanidin-3,5-O-diglucoside, luteolin-7-O-rhamnoside, cyanidin-3-O-(6″-O-p-Coumaroyl)glucoside, kaempferol-3-O-rutinoside, 3,5,7-trihydroxyflavanone, quercetin-3-O-rutinoside, delphinidin-3,5,3′-tri-O-glucoside, naringenin chalcone, quercetin-3-O-glucoside, which showed positive correlations; *UGT77B2* showed positive correlations with apigenin-8-C-glucoside and apigenin-6-C-glucoside; *3GT* showed positive correlations with delphinidin-3,5,3′-tri-O-glucoside and cyanidin-3,5-O-diglucosid; *4CL* showed positive correlation with quercetin-3-O-glucoside and butin; and *3AT* showed negative correlation with delphinidin-3-O-(6″-O-p-coumaroyl) glucoside. Based on the above analysis, these five genes (*CHS2*, *UGT77B2*, *3GT*, *4CL*, *3AT*) were highly correlated with DAFs, and it was hypothesized that these might be involved in regulating the biosynthesis of related metabolites.

### 3.7. Transcription Factor-Mediated Analysis of Flavonoid Biosynthesis

In addition to key structural genes, transcription factors (TFs) involved in the regulation of the biosynthetic pathway are indispensable. To further explore the key TFs involved in flavonoid biosynthesis, we annotated all transcript sequences with TFs using the PlantTFDB database. In this study, we conducted transcriptome sequencing to identify 890 TFs from 42 gene families. Among these, we noted a total of 180 TFs differentially expressed (FPKM ≥ 10 in all samples). Our results revealed that these 180 TFs belonged to 30 gene families, with the SAP gene family being the most abundant, followed by bZIP, GRAS, ERF, MYB, Trihelix, ARF, bHLH and others (Supplementary Figure S5). These TFs are also the most likely to regulate flavonoid biosynthesis in *D. devonianum*.

To comprehensively investigate the TFs closely associated with flavonoid biosynthesis in *D. devonianum* stems, 180 TFs were correlation analyzed with the above 47 flavonoid structural genes, and a correlation network interplay diagram was constructed (Figure 5C). The results revealed significant correlations between 82 TFs (Supplementary Table S9) and 42 flavonoid biosynthesis structural genes (|R| ≥ 0.8, *p < 0.01*). Notably, bHLH5 and bHLH4 exhibited positive correlations with *F3H2* and *CYP75A*, while bHLH3 showed a positive correlation with *3AT1*. Additionally, MYB1 displayed a positive correlation with *5GT4*, MYB2 was positively correlated with *FG2*, whereas NF-YC3 and bHLH19 were negatively correlated with *4CL3* and *3AT2*. In conclusion, my results emphasize that bHLH3, bHLH4, bHLH5, MYB1 and MYB2 may interact with flavonoid biosynthesis structural genes and participate in the regulation of flavonoid synthesis.

### 3.8. Weighted Gene Co-Expression Network Analysis

**Figure 6:**
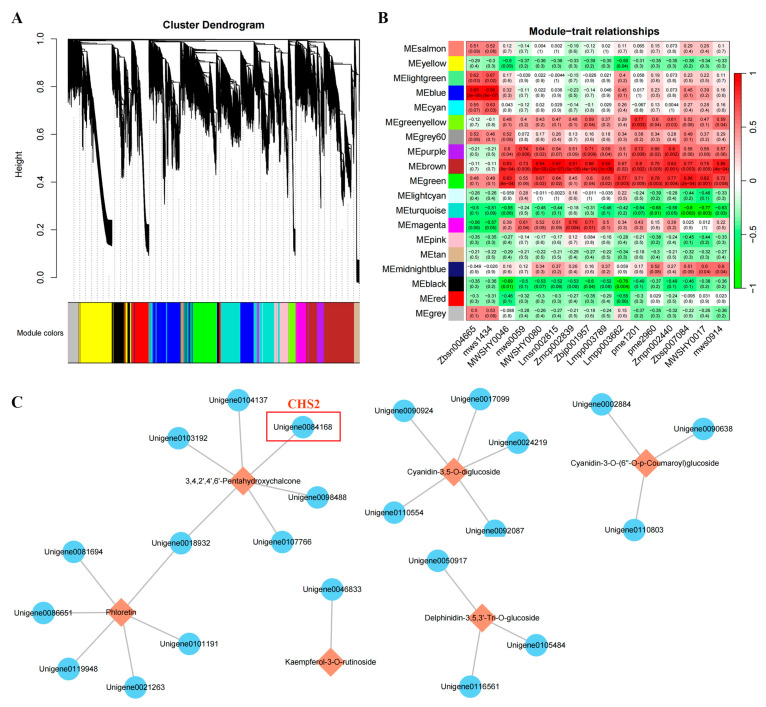
WGCNA results of *D. devonianum* stem genes and accumulation of flavonoid metabolites: (**A**) WGCNA co-expression hierarchical clustering tree (19 gene expression modules, different colors represent different gene modules). (**B**) Correlation analysis of the 19 gene modules with 16 flavonoid metabolites (the value in each box indicates the Pearson’s correlation coefficient between the modules and metabolites, and each bracketed value indicates the *p*-value; the color scale on the right side indicates the degree of correlation between the module and the metabolite, red color indicates high correlation). (**C**) Correlation network diagram of DEGs with flavonoid metabolites (orange boxes indicate metabolites, blue circles indicate DEGs).

To further investigate the gene regulatory network in the flavonoid biosynthesis pathway in *D. devonianum* stems, we integrated metabolomic and transcriptomic data for weighted gene co-expression network analysis (WGCNA). The metabolome was screened for 16 significantly different metabolites annotated to flavonoid metabolite-related synthetic pathways that were used for association analysis, and the remaining 23,864 genes filtered from the transcriptomic data were used to construct a topological overlap matrix and perform hierarchical clustering analysis, resulting in the identification of 19 gene modules (Figure 6A). Association analysis of modules with metabolites revealed diverse correlations between flavonoid metabolites and individual gene modules (Figure 6B). We found that the MEbrown module was highly correlated with the content of all 16 flavonoid metabolites. Based on the correlation queue between modules and metabolites (MMbrown ≥ 0.8, R ≥ 0.98 and *p < 0.01*), 24 DEGs were identified in the brown module, and co-expression network construction was performed (Figure 6C). The results showed that flavonoid metabolites were positively regulated by one or more genes. Among them, phloretin and 3,4,2′,4′,6′-Pentahydroxychalcone were positively regulated by six genes, respectively, followed by cyanidin-3,5-O-diglucoside, which was positively regulated by five genes. Cyanidin-3-O-(6″-O-p-Coumaroyl) glucoside and delphinidin-3,5,3′-Tri-O-glucoside were positively regulated by three genes each, and finally, kaempferol-3-O-rutinoside was positively regulated by one gene. Notably, related regulatory genes for cyanidin-3-O-(6″-O-p-Coumaroyl) glucoside, delphinidin-3,5,3′-Tri-O-glucoside and cyanidin-3,5-O-diglucoside were successfully screened out, which may lead to the color difference between the RDd and GDd. Our results indicated that these 24 genes play important regulatory roles in the flavonoid metabolite biosynthesis pathway.

In addition, the green module showed a high correlation with flavonoid metabolites, the genes were narrowed down based on MMgreen ≥ 0.8 and p.MMgreen ≤ 0.05, and the remaining 1804 genes were screened for further analysis. The remaining 1848 genes were screened for further analysis by narrowing down the genes based on MMblue ≥ 0.8 and p.MMblue ≤ 0.05 in the blue module. The genes in the brown, green and blue modules were combined and analyzed with transcriptional metabolic association analysis, and eight genes were finally screened (Supplementary Table S10). Notably, *CHS2*, *4CL* and *UGT77B2* were screened.

### 3.9. Mapping of Key Genes in the Flavonoid Biosynthetic Pathway

**Figure 7:**
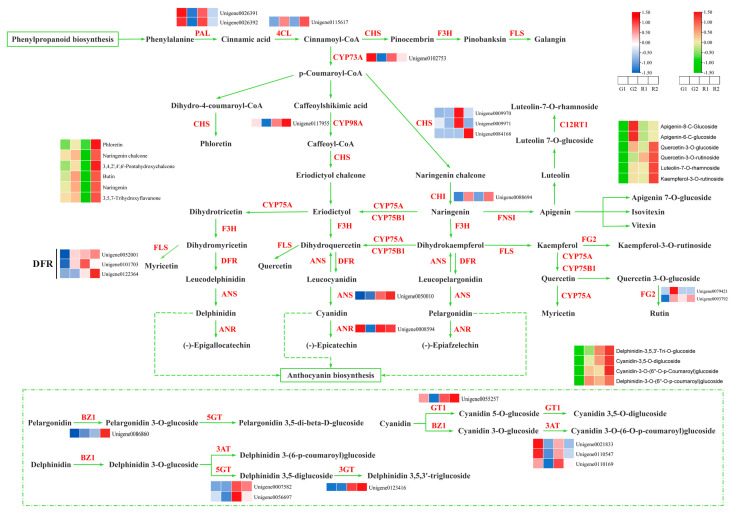
Dynamic expression analysis of genes in the flavonoid biosynthesis pathway of *D. devonianum*. (*PAL*: Phenylalanine ammonia-lyase; *4CL*: 4-coumarate--CoA ligase; *CYP73A*: Trans-cinnamate 4-monooxygenase; *CYP98A*: 5-O-(4-coumaroyl)-D-quinate 3′-monooxygenase; *CHS*: Chalcone synthase; *CHI*: Chalcone isomerase; *DFR*: Flavanone 4-reductase; *ANS*: Anthocyanidin synthase; *ANR*: Anthocyanidin reductase; *FG2*: Flavonol-3-O-glucoside L-rhamnosyltransferase; *BZ1/UGT77B2*: Anthocyanidin 3-O-glucosyltransferase; *5GT*: Anthocyanidin 3-O-glucoside 5-O-glucosyltransferase; *3GT*: Anthocyanin 3′-O-beta-glucosyltransferase; *GT1*: Anthocyanidin 5,3-O-glucosyltransferase; *3AT*: Anthocyanidin 3-O-glucoside 6″-O-acyltransferase).

In order to visualize the expression of flavonoid biosynthesis pathway genes in RDd and GDd, 24 key genes regulating flavonoid biosynthesis, anthocyanin biosynthesis, and flavonoid and flavonol biosynthesis pathways were screened. Based on the 24 key enzyme encoding genes for flavonoid biosynthesis and the KEGG synthesis pathway (ko00941, ko00942, ko00944), we mapped the key regulatory pathways of flavonoid biosynthesis in *D. devonianum* (Figure 7). The expression of *PAL*, *4CL*, *CHS*, *DFR*, *CHI*, *ANS*, *UGT77B2*, *5GT*, *3GT*, *GT1* varied significantly. *PAL* and *4CL* were involved in phenylpropanoid biosynthesis upstream of flavonoid biosynthesis, and the expression of *PAL* showed a tendency to be down-regulated with stem maturation, whereas the expression of *4CL* showed an up-regulation trend with stem maturation. *CHS* expression is up-regulated in RDd, but the trend varies. The *DFR* family is commonly expressed at maturity and is higher in RDd than in GDd. *ANS* was significantly up-regulated in RDd, *UGT77B2* was expressed in R2, and the *5GT*, *3GT*, and *GT1* showed a significant up-regulation in the expression pattern in RDd. We hypothesize that low expression of *CHS* and *DFR* in the GDd inhibits flavonoid synthesis, while low expression of *GT1* inhibits pigment accumulation, differentiating the color from that of the RDd.

Meanwhile, simultaneous analysis of the accumulation trend of metabolites via flavonoid biosynthesis and gene expression trend showed that the expression trend of the annotated *CHS* family was basically consistent with its catalyzed products naringenin chalcone and phloretin. The accumulation pattern of *CHI* and its catalytic substrates naringenin, quercetin-3-O-glucoside and luteolin-7-O-rhamnoside was basically the same. No genes consistent with the accumulation pattern of apigenin-8-C-glucoside apigenin-6-C-glucoside were identified, and the gene that catalyzes this step may not have been annotated. The expression of *FG2* was essentially identical to that of kaempferol-3-O-rutinoside and quercetin-3-O-rutinoside. *3GT* is consistent with the accumulation pattern of delphinidin-3,5,3′-Tri-O-glucoside, and *GT1* is essentially identical to cyanidin-3,5-O-diglucoside. The inconsistent expression pattern of *3AT* with its catalyzed substrate could be the involvement of this gene in other pathways. The other pathway genes did not reflect a clear pattern of correlation with compound accumulation, and their functions should be confirmed in the context of specific experiments.

*3.10. qRT-PCR Validation of RNA-seq Data*

**Figure 8:**
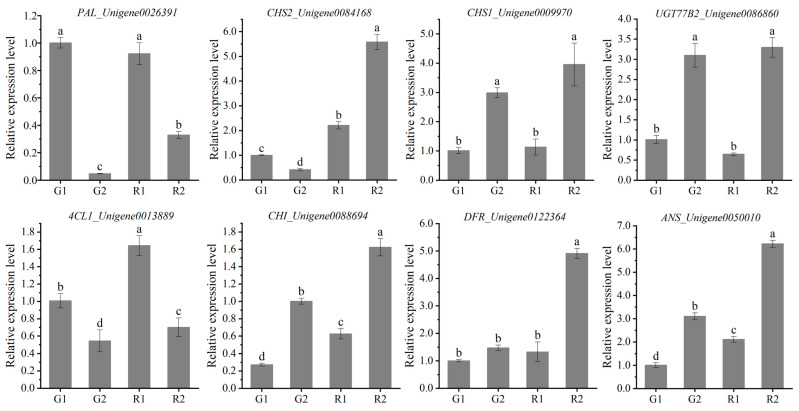
Expression analysis of key genes of flavonoid biosynthesis pathway. Values are mean ± SD (*n* = 3 independent measurements); different lowercase letters (a, b, c, d) indicate significant differences, *p* < 0.05.

To verify the accuracy of the transcriptome data, eight DEGs in the regulatory flavonoid metabolic pathway were selected and validated using real-time fluorescence quantification (qRT-PCR). The results showed that the qRT-PCR gene expression trends of *PAL*, *CHS2*, *4CL1*, *CHI*, *DFR* and *ANS* were generally consistent with the transcriptome expression trends, indicating that the transcriptome data were valid and reliable (Figure 8).

## 4. Discussion

*D. devonianum* contains a wide variety of flavonoid, which, along with polysaccharides, are key bioactive components of the plant, exhibiting various pharmacological activities such as anti-inflammatory and antioxidant activities [38]. Currently, research on *D. devonianum* has primarily focused on cultivation [39,40] and polysaccharide extraction [41,42]. In this study, 244 flavonoid metabolites were identified in GDd and RDd using UPLC-MS, which were mainly flavones and flavonols. Flavones had the highest relative content in G2. Flavonols and anthocyanidins had the highest relative content in R2. We screened 38 DAFs across four comparison groups, with 16 of these annotated to the flavonoid biosynthesis pathway. Notably, the accumulation patterns of these metabolites differed significantly in GDd and RDd. Further analysis revealed that the majority of flavonoid metabolite in *D. devonianum* exists in the form of glucoside. The variations in flavone metabolites were concentrated in the glycosides formed by tricin, luteolin and apigenin, and the variations in flavonol metabolites were concentrated in the glycosides formed by kaempferol, quercetin and isorhamnetin, which were basically the same as the compositions of those found in the leaves and roots of *Sarcandra glabra* [43]. In addition, the variations in anthocyanidin metabolites were concentrated in the glycosides formed by cyanidin and delphinidin, which is basically consistent with the results of the quantitative assessment of the anthocyanin composition types of the green and red stems of *Dendrobium candidum* [44]. In this experiment, specific anthocyanidins such as cyanidin-3,5-O-diglucoside delphinidin-3,5,3′-Tri-O-glucoside and cyanidin-3-O-(6″-O-p-Coumaroyl)glucoside can be used as characteristic metabolites of different colored stems of *D. devonianum*.

High-throughput sequencing and multi-omics technologies can provide dynamic information on plant growth and development at the system and cellular level. For example, the researchers used widely targeted metabolomic and transcriptomic approaches to study the dynamic changes in key flavor components in samples from various processing steps of Tieguanyinoolong tea production investigated to investigative the formation of characteristic flavors during the production of Tieguanyin [45]. The physiological mechanism of flesh browning in apple fruit during cold storage was revealed by analyzing transcriptomic and metabolomic parameters [46]. The molecular mechanisms underlying the differential accumulation of anthocyanins in mango pericarps under light and shade conditions were explored by a combined multi-omics analysis approach [47]. Comprehensive metabolomics and transcriptomics analyses elucidate the regulatory mechanism for the synthesis of flavonoids medicinally active components of black wolfberry [48]. In this study, the KEGG enrichment pathways of DAFs and DEGs were mainly flavonoid biosynthesis, anthocyanin biosynthesis, and flavone and flavonol biosynthesis when GDd was compared with RDd, further demonstrating the importance of the flavonoid synthesis pathway in stem color changes. The flavonoid and anthocyanin pathways are closely related to stem color formation, and their synthetic pathways are controlled by a series of structural genes. Based on the flavonoid synthesis pathway and transcriptomics data of *D. devonianum* stem samples, 47 flavonoid synthesis pathway-related genes were identified and screened, regulating 21 enzymes, which are basically similar to the genes of the flavonoid biosynthesis pathway in the stem of *Dendrobium officinale* [49]. Among them, the expression of *CHS2*, *UGT77B2*, *3GT* and *4CL* were higher in RDd than in GDd and showed a significant positive correlation with flavonoid metabolites. *3AT* was higher in GDd than in RDd and showed a negative correlation with flavonoid metabolites. We hypothesized that these five genes might be key genes involved in regulating the accumulation of flavonoid metabolites.

TFs play a key role in regulating the expression of structural genes involved in the flavonoid biosynthesis pathway, with MYB, bHLH and WD40 b TF families being the primary. For instance, a study on lily cultivar “Vivian” identified four bHLH genes involved in regulating flavonoid biosynthesis [50]. A MYB gene and a bHLH gene in *Dendrobium* hybrid orchids were discovered, and DhMYB2 interacted with DhbHLH1 to modulate anthocyanin production in *Dendrobium* hybrid petals [51]. In this study, a total of 890 TFs were identified from *D. devonianum* stems, with 180 TFs belonging to 30 gene families selected based on FPKM ≥ 10 across all samples. Among these families, SAP TFs were the most abundant, followed by bZIP, bHLH and MYB. It is worth noting that no WD40 TFs were detected in our analysis, suggesting that bHLH and MYB are the primary regulatory TF families regulating flavonoid biosynthesis in *D. devonianum* stems. Correlation analysis of TFs with flavonoid structural genes revealed significant associations between 82 TFs and 42 structural genes involved in flavonoid biosynthesis (|R| ≥ 0.8, *p < 0.01*). Specifically, DdbHLH3, DdbHLH4 and DdbHLH5 exhibited positive correlations with *3AT1*, *CYP75A* and *F3H2*, respectively. DdMYB1 and DdMYB2 were positively correlated with *5GT4* and *FG2*, respectively. These findings suggest potential interactions among DdbHLH3, DdbHLH4, DdbHLH5, DdMYB1 and DdMYB2 might be related to flavonoid biosynthesis structural genes interacting with each other and thus regulating flavonoid synthesis.

To provide stronger support for the process of flavonoid biosynthesis in the *D. devonianum* stem, we employed WGCNA to identify key genes regulating 16 flavonoid metabolites. From this, we selected 24 highly correlated genes (R ≥ 0.98, *p < 0.01*) to construct correlation network maps. Several flavonoid biosynthesis pathway genes that may be closely related to flavonoid metabolites in *D. devonianum* were identified, including *CHS2*, *CHI*, *4CL*, *GT1*, *UGT77B2*, etc. The functional genes in the flavonoid biosynthesis pathway are genes that regulate enzymes in the metabolic pathway between them, such as *PAL*, *CHS*, *CHI*, *F3H*, *FLS*, *ANS*, etc. In recent years, these important functional genes have been cloned in many plants, and the mechanisms of action of some of the proteins encoded by them have been gradually revealed [52]. *CHS,* as the first key node in the flavonoid biosynthesis pathway, showed the same expression pattern as its catalyzed products, naringenin chalcone and phloretin, in this study, and there was a high correlation between *CHS2* and the metabolites. Expression of the *CHS2* led to the accumulation of naringenin chalcone and phloretin, which in turn enhanced the accumulation of anthocyanins and other substances in the flavonoid biosynthesis pathway. In previous studies, it has been demonstrated that *CHS* expression affects the accumulation of flavones and anthocyanins [53,54]. *CHI* is a key enzyme gene regulating naringenin synthesis, and changes in its activity affect the accumulation of flavonoid metabolites [55]. In this study, the expression of *CHI* in RDd caused it to accumulate more flavonoid metabolites than GDd. *UGT77B2*, *3GT* and *GT1,* as key regulatory genes of anthocyanin biosynthesis [56], exhibit the same accumulation pattern as cyanidin-3-O-(6″-O-p-Coumaroyl) glucoside, delphinidin-3-O-(6″-O-p-coumaroyl) glucoside, delphinidin-3,5,3′-Tri-O-glucoside and cyanidin-3,5-O-diglucoside. The expression of related genes catalyzed the accumulation of cyanidin and delphinidin glycosides. Therefore, the changes in metabolites in the anthocyanin biosynthesis are mainly the changes in glycosides. Delphinidin related metabolites are key substances in the purple tone of color, while cyanidin induces red color. In this study, the accumulation of cyanidin and delphinidin glycosides resulted in a purplish-red appearance of *D. devonianum* stems, which is consistent with the predominant chromogenic species in purple-red peony [57]. In addition, we performed RT-qPCR validation on the transcriptome data, and the results were consistent with the RNA-seq, confirming the accuracy of our transcriptome analysis.

## 5. Conclusions

In this study, the *D. devonianum* green and purplish-red stems (GDd and RDd) were used as the materials to reveal the flavonoid metabolites formation mechanism based on the variation pattern of flavonoid metabolites at different growth stages. The content and types of flavones and flavonols in *D. devonianum* stems were the highest. Cyanidin and delphinidin were the major anthocyanidins, with cyanidin-3-O-(6″-O-p-Coumaroyl) glucoside and delphinidin-3-O-(6″-O-p-coumaroyl) glucoside being the highest relative content in the RDd. Sixteen significant differential metabolites were identified. The differential transcriptional expression of key structural genes involved in the flavonoid synthesis pathway is the main reason for the different flavonoid metabolites in *D. devonianum* stems. Based on correlation analysis, we screened five genes regulating the accumulation of flavonoid metabolites, and five key TFs that might be involved in the regulation of flavonoid structural genes were found. A co-analysis of transcriptional and metabolism based on WGCNA finally screened eight key candidate genes involved in flavonoid biosynthesis. This study provides a basis for the regulation of flavonoid biosynthesis in *D. devonianum* and the functional identification of key genes, as well as a new idea for the development of pharmacologically active ingredients in *D. devonianum.*

## Data Availability

The data supporting the findings of this manuscript are available from the corresponding authors upon reasonable request.

## Supplementary Materials

The following supporting information can be downloaded at: https://www.mdpi.com/article/10.3390/cimb46120855/s1, Table S1: Real-time PCR primer sequences; Table S2: Different flavonoid metabolites in the stem of *D. devonianum*; Table S3: Validation parameters of PLS-DA and OPLS-DA models for each comparison group; Table S4: Differential accumulation metabolites; Table S5: Metabolites identified by the flavonoid biosynthetic pathway; Table S6: Statistical analysis of transcriptome sequencing quality in two periods of GDd and RDd; Table S7: Shared differential gene log2(fc) values; Table S8: Expression of structural genes related to flavonoid biosynthesis pathway; Table S9: Transcription factors of different TFs families; Table S10: Screening of key genes for flavonoid biosynthesis in *D. devonianum.* Figure S1: (A) Classification statistics of identified broad-targeted metabolites. (B) Relative content of anthocyanidins in different samples. (C) PCA Analysis; Figure S2: KEGG enrichment pathway of DAFs; Figure S3: Differential Metabolite Statistical Analysis Chart; Figure S4: Correlation analysis of transcriptome data; Figure S5: Statistics of the number of gene families of TFs.
