# Supplementary material for: Molecular and Metabolic Regulation of Flavonoid Biosynthesis in Two Varieties of Dendrobium devonianum
Source: Curr Issues Mol Biol. 2024 Dec 18;46(12):14270–90. doi: 10.3390/cimb46120855 (PMC11674678; doi:10.3390/cimb46120855)
Supplement: Supplementary file 1 [file cimb-46-00855-s001.zip › cimb-3259418-supplementary.pdf]

Supplemental files

Table S1. Real−time PCR primer sequences.

Gene name	Gene ID	Primer sequence（5′→3′）
PAL	Unigene0026391	F：CGGAGAGAAGGTGGCTTCG<br>R：CAAGCACTCGAACAGGGGAT
4CL1	Unigene0013889	F：GGAGATGGCGAAGAGTGAGG<br>R：AGCTCGGCATTCCTAACGAC
CHS	Unigene0009970	F：CATCGGTAGGGCAAACCCAG<br>R：GGCGCTTTCTGATTGCTGTC
CHS2	Unigene0084168	F：TTGGCGGAATACGGGAACAT<br>R：ACAGTAATTCCCGGGCCAAG
CHI	Unigene0088694	F：TTCCCCTAGCGGCACTCTAA<br>R：AACTTGCTCCAACTTGCCCT
DFR	Unigene0122364	F：TGAGCATCCCAAAGCAAATGG<br>R：TTTGTTAAGTGCTTACTTCTGAGG
UGT77B2	Unigene0086860	F：GATGTTGTGCAGGCCGATCT<br>R：AACTCCATTCATGGCTCCAACA
ANS	Unigene0050010	F：CGAGGGAGAAGGTTGTGCTT<br>R：TGTCTCCACTTCATGCCACC

Table S2. Different flavonoid metabolites in the stem of *D. devonianum.*

Compounds	Class II	Compounds	Class II
6,8-Dihydroxy-2-(2-hydroxy-4-methoxybenzyl)-7-methyl-3,4-dihydronaphthalen-1(2H)-one	Other Flavonoids	Aromadendrin-7-O-glucoside	Flavanonols
Isovitexin-2''-O-xyloside	Flavones	Quercetin-3-rutinoside-7-galactoside	Flavonols
Delphinidin-3-O-rutinoside-7-O-glucoside	Anthocyanidins	Quercetin-3-O-apiosyl (1→2) galactoside	Flavonols
5-Hydroxy-7,4'-dimethoxy-6,8-dimethylflavone	Flavanones	Apigenin-7-O-(6''-malonyl)-glucoside	Flavones
2,6-Dimethoxypydroquinone-1-O-glucoside	Other Flavonoids	Rhamnetin-3-O-Rutinoside	Flavonols
Tricin-7-O-(2''-feruloyl)-glucuronide	Flavones	Isorhamnetin-3-O-neohesperidoside	Flavonols
Kaempferol-3-O-robinobioside	Flavonols	3'-methoxyquercetin-3-O-L-rhamnosyl (1→2)-glucopyranoside	Flavonols
4-C-Glucose-1,3,6-trihydroxy-7-methoxyxanthone	Other Flavonoids	Eriodictyol-7-O-Rutinoside	Flavanones
5-hydroxy-3-(2-hydroxy-4-methoxybenzyl)-7-methoxychroman-4-one	Other Flavonoids	2',4'-dihydroxy-4-methoxydihydrochalcone	Chalcones
Vitexin-2''-O-rhamnoside	Flavones	Hesperetin-7-O-glucoside	Flavanones
5,7,2'-Trihydroxy-8-methoxyflavone	Flavones	Isorhamnetin-7-O-glucoside	Flavonols
Quercetin-3-O-rutinoside	Flavonols	Quercetin-7-O-glucoside	Flavonols
Quercetin-3-O-(4''-O-glucosyl)-rhamnoside	Flavonols	Gossypetin-3-O-rutinoside-8-O-glucoside	Flavonols
Genistein-7-O-Glucoside	Isoflavones	Isorhamnetin-3-O-sophoroside-7-O-rhamnoside	Flavonols
Apigenin-7-O-glucoside	Flavones	5,7,3',4'-Tetrahydroxyflavanone	Flavanones
Chrysin-7-O-(6'-malonyl)-glucoside	Flavones	Apigenin-6-C-xyloside-8-C-arabinoside	Flavones
6,7-dihydroxy-1,3-dimethoxyxanthen-9-one	Other Flavonoids	Dihydromyricetin-3-O-glucoside	Flavanonols
Chrysoeriol glucosyl xylosyl glucoside	Flavones	Viscidulin III	Flavones

Naringenin (5,7,4'-Trihydroxyflavanone)	Flavanones	Calyxanthone	Other Flavonoids
Kaempferol-3-O-(2''-O-acetyl)-glucuronide	Flavonols	Norartocarpetin	Flavones
5,7-Dihydroxy-6,8-dimethyl-3-(2',4'-hydroxybenzyl)-chroman-4-One	Other Flavonoids	Epicatechin gallate	Flavanols
Quercetin-3-O-galactoside	Flavonols	Apigenin-6-C-glucose-8-C-rhamnoside	Flavones
Butin; 7,3',4'-Trihydroxyflavanone	Flavanones	Rhamnazin 3-Glucosyl-Arabinofuranoside	Flavonols
Vitexin-7-O-glucoside	Flavones	Morin-3-O-lyxoside	Flavonols
(2S)-Abyssinone II	Flavanones	Morin-3-O-arabinoside	Flavonols
Naringenin chalcone; 2',4,4',6'-Tetrahydroxychalcone	Chalcones	Apigenin-6,8-di-C-arabinoside	Flavones
Vitexin-2''-O-galactoside	Flavones	Artocarpanone	Flavanones
Tricin-4'-O-syringic acid	Flavones	Odoricarpan	Flavones
Naringenin-7-O-(6''-malonyl)-glucoside	Flavanones	Gnetifolin B	Flavonols
Phloretin	Chalcones	4',5,6,7,8-Pentamethoxyflavone	Flavones
1,2,5,7,8-pentahydroxy-3-methylanthracene-9,10-dione	Other Flavonoids	Dihydromarein	Chalcones
Aracarpene 1	Isoflavones	Galangin-7-O-glucoside	Flavonols
Apigenin-6,8-di-C-glucoside	Flavones	Pectolinarigenin-7-O-glucoside	Flavones
3'-O-methylorobol	Isoflavones	Genistein-7-O-galactoside	Isoflavones
8-Methoxyapigenin	Flavones	Luteolin-3'-O-glucoside	Flavones
Delphinidin-3,5,3'-Tri-O-glucoside	Anthocyanidins	Kaempferol-3-O-galactoside	Flavonols
Aracarpene 2	Isoflavones	Swertiajaponin	Flavones
Chrysoeriol-7-O-(6''-malonyl)-glucoside	Flavones	Apigenin-6-C-glucoside	Flavones
6-C-MethylKaempferol-3-glucoside	Flavonols	Apigenin-6-C-arabinoside-8-C-xyloside*	Flavones
Genistein	Isoflavones	Quercetin-3,7-Di-O-glucoside	Flavonols
Apigenin; 4',5,7-Trihydroxyflavone	Flavones	Schaftoside	Flavones
Hesperetin-7-O-(6''-malonyl)-glucoside	Flavanones	Dihydrokaempferol-3-O-glucoside	Flavanonols
Homeriodictyol	Flavanones	6,8-Dimethyl-5,7,4'-trihydroxyflavanone	Flavanones
Quercetin-4'-O-glucoside	Flavonols	Naringenin-7-O-Rutinoside-4'-O-glucoside	Flavanones
Quercetin-3-O-robinobioside	Flavonols	Naringenin-4'-O-glucoside	Flavanones
Tricin-7-O-(2''-Sinapoyl)-glucoside	Flavones	6-Hydroxykaempferol-3,6,7-O-triglucoside	Flavonols
Naringenin-7-O-Rutinoside	Flavanones	Naringenin-7-O-Neohesperidoside	Flavanones
Kaempferol-3-O-rutinoside	Flavonols	Blumeatin	Flavanones
Eriodictyol-7-O-glucoside	Flavanones	Genistein-7-O-(6''-malonyl)-glucoside	Isoflavones
Okanin-3'-O-β-D-glucoside	Chalcones	Chrysoeriol-5,7-di-O-glucoside	Flavones
5,6,7,3',4',5'-hexanmethoxyflavone	Flavones	Quercetin-3-O-glucoside	Flavonols
4-Hydroxychalcone	Chalcones	Quercetin-5-O-β-D-glucoside	Flavonols
Isorhamnetin-3-O-glucoside-7-O-rhamnoside	Flavonols	Quercetin-3-O-rhamnoside	Flavonols
Morin	Flavonols	Tamarixetin-3-O-rutinoside	Flavonols
1,2,4,5,8-pentahydroxy-6-methylanthracene-9,10-dione	Other Flavonoids	3,5,3′,4′,5′-penta-hydroxyflavan-7-gallate	Flavanols
Pinocembrin-7-O-(6''-O-malonyl)-glucoside	Flavanones	Epigallocatechin-3-O-gallate	Flavanols
Quercetin-3-O-rutinoside-7-O-glucoside	Flavonols	Gallocatechin gallate	Flavanols
Gossypetin-3-O-rutinoside-8-O-rhamnoside	Flavonols	Eriodictyol-8-C-glucoside	Flavanones
Tricetin-5-O-(6''-malonyl)-glucoside	Flavones	Jaceosidin-7-O-Glucoside	Flavones
5,7,4'-Trihydroxy-8-methoxyflavone-6-C-[Xylosyl-(1-2)]-glucoside	Flavones	Protogenkwanone	Other Flavonoids
Quercetin-7-O-rutinoside-4'-O-glucoside	Flavonols	Taxifolin (Dihydroquercetin)	Flavanonols

Apigenin-8-C-Glucoside	Flavones	5,7,4'-Trihydroxy-3',5'-dimethoxyflavone	Flavones
Dihydrokaempferol-7-O-glucoside	Flavanonols	Yuanhuanin	Flavones
Myricetin-3-O-rhamnoside	Flavonols	Tricin-7-O-saccharic acid	Flavones
Hispidulin-8-C-glucoside	Flavones	Cyanidin-3-O-(6''-O-p-Coumaroyl)-glucoside	Anthocyanidins
6''-O-Malonylgenistin	Isoflavones	Luteolin-7-O-(6''-malonyl)-glucoside*	Flavones
Orientin-6-C-arabinoside	Flavones	Luteolin-4'-O-glucoside	Flavones
Maesopsin	Other Flavonoids	Vitexin-2''-O-glucoside	Flavones
3,5,7-Trihydroxyflavone	Flavonols	Taxifolin-2-O-glucoside	Flavanonols
3,4,2',4',6'-Pentahydroxychalcone	Chalcones	Isohemiphloin	Flavanones
Naringenin-7-O-glucoside	Flavanones	6-Hydroxykaempferol-3,6-O-Diglucoside	Flavonols
Luteolin-7-O-glucoside	Flavones	Isoschaftoside	Flavones
Kaempferol-4'-O-glucoside	Flavonols	4'-O-Glucosylvitexin	Flavones
Butin-7-O-glucoside	Flavanones	Sexangularetin-3-O-glucoside-7-O-rhamnoside	Flavonols
Phloretin-2'-O-(6''-O-acetyl)-glucoside	Chalcones	Hydroxy isoliquiritigenin glucoside	Chalcones
Quercetin-3-O-[rhamnosyl(1→2) glucosyl]-5-O-glucoside*	Flavonols	Isovitexin-2''-O-rhamnoside	Flavones
3',4',5',5,7-Pentamethoxyflavone	Flavones	Rhamnosyl Phellamurin	Flavanonols
kaempferol-3-caffeoyldiglucoside	Flavonols	Quercetin-3-O-sambubioside	Flavonols
6,7,8-Tetrahydroxy-5-methoxyflavone	Flavones	Apigenin-5-O-glucoside	Flavones
Laricitrin-3-O-glucoside	Flavonols	Isorhamnetin-3-O-rutinoside	Flavonols
Mearnsetin-3-O-glucoside	Flavonols	6-C-Methylquercetin-3-O-rutinoside	Flavonols
Rhamnetin; 3,5,3',4'-Tetrahydroxy-7-Methoxyflavone	Flavonols	Limocitrin-3-O-galactoside	Flavonols
Cyanidin-3-O-(6''-O-sinapoyl) sophoroside-5-O-glucoside	Anthocyanidins	Hispidulin-7-O-glucoside	Flavones
Drimiopsin C	Other Flavonoids	Delphinidin-3-O-(6''-O-p-coumaroyl)-glucoside	Anthocyanidins
Isovitexin-4'-O-glucoside	Flavones	Kaempferol-3-O-glucoside-7-O-rhamnoside	Flavonols
3,5,7-Trihydroxyflavanone	Flavanonols	Catechin gallate	Flavanols
2',3',4',5,7-Pentahydroxyflavone	Flavones	Hesperetin-5-O-glucoside	Flavanones
Phloretin-4'-O-glucoside	Chalcones	Genkwanin-6-C-(2''-O-apiosyl)-glucoside	Flavones
Butein	Chalcones	6-Methoxykaempferol-3-O-glucoside	Flavonols
Chrysoeriol-7-O-gentiobioside	Flavones	Kaempferol-3-O-sophorotrioside	Flavonols
Kaempferol-3-O-(6''-Rhamnosyl-2''-Glucosyl)-Glucoside	Flavonols	Apigenin-6-C-(2''-glucuronyl) xyloside	Flavones
Isosalipurposide	Chalcones	Vicenin-2,6''-O-Glucoside	Flavones
5,7,3'-Trihydroxy-4'-methoxyflavone	Flavones	Tricin-7-O-(6''-O-malonyl)-glucoside	Flavones
3,5,4'-Trihydroxy-7-methoxyflavone	Flavonols	Kaempferol-3-O-(6''-malonyl)-glucoside	Flavonols
5,6,7-Tetrahydroxy-8-methoxyflavone	Flavones	Calycosin-7-O-glucoside	Isoflavones
Munduleaflavanone B	Flavanones	Kaempferol-7-O-glucoside	Flavonols
6-Hydroxyluteolin 5-glucoside	Flavones	Carthamone	Chalcones
Sophoricoside	Isoflavones	Hesperetin-3'-O-glucoside	Flavanones
Gallocatechin 3-O-gallate	Flavanols	Isohyperoside	Flavonols
Glucosyl Amurensin	Flavonols	Quercetin-3-O-α-L-arabinofuranoside	Flavonols
Apigenin-8-C-(2''-glucosyl)-arabinoside	Flavones	Kaempferol-3-O-neohesperidoside	Flavonols
Hesperetin-7-O-rutinoside	Flavanones	Diosmetin-6-C-glucoside	Flavones
Cyanidin-3,5-O-diglucoside	Anthocyanidins	Luteolin-7-O-neohesperidoside	Flavones
Apigenin-4'-O-glucoside	Flavones	Quercetin-3-O-sophoroside	Flavonols
Genistein-8-C-glucoside	Isoflavones	Naringenin-6-C-Glucoside	Flavanones
Dihydrocharcone-4'-O-glucoside	Chalcones	Quercetin-3-O-xyloside	Flavonols

Methylhesperidin	Flavanones	Cyanidin-3-O-(6''-O-malonyl)-glucoside-5-O-glucoside	Anthocyanidins
Kaempferol-3-O-(6''-malonyl) galactoside	Flavonols	Sterubin 5-O-Glucoside	Flavanones
Isobavachalcone glucoside	Chalcones	Chrysoeriol-6,8-di-C-glucoside	Flavones
2-(3,4-dihydroxyphenyl)-5-hydroxy	Flavonols	6-Hydroxykaempferol-7-O-glucoside	Flavonols
Isovitexin-7-O-glucoside	Flavones	Isorhamnetin-3-O-Glucoside	Flavonols
6-Hydroxykaempferol-3,7,6-O-triglycoside	Flavonols	Apigenin-7-O-(2''-apiosyl) glucoside	Flavones
Myricetin-3-O-galactoside-3'-O-rhamnoside	Flavonols	Kaempferol-3-O-sophoroside-7-O-rhamnoside	Flavonols
Kaempferol-3-O-(2''-sinapoyl) glucosyl-(1→2) - (6''-malonyl) glucoside	Flavonols	Luteolin-7-O-rutinoside	Flavones
Apigenin-6-C-(2''-glucosyl) arabinoside	Flavones	Isorhamnetin-3-O-arabinoside-7-O-rhamnoside	Flavonols
Apigenin-6-C-(2''-xylosyl) glucoside	Flavones	Petunidin-3-O-(6''-O-p-Coumaroyl) glucoside	Anthocyanidins
Catechin	Flavanols	Genistein-7-O-galactoside-rhamnose	Isoflavones
Hispidulin-8-C-(2''-O-glucosyl) glucoside	Flavones	Apigenin-7-O-rutinoside	Flavones
Violanthin	Flavones	Luteolin-6-C-(2''-glucuronyl) glucoside	Flavones
Meratin	Flavonols	6-Hydroxykaempferol-3-O-rutinoside-6-O-glucoside	Flavonols
Kaempferol-3-O-neohesperidoside-7-O-glucoside	Flavonols	Kaempferol-3-O-(2''-feruloyl) glucosyl-(1→2) -(6''-malonyl) glucoside	Flavonols
Phloretin-2'-O-glucoside	Chalcones	Hispidulin-8-C-(2''-O-xylosyl) xyloside	Flavones

Table S3. Validation parameters of PLS−DA and OPLS−DA models for each comparison group.

Group	Model	pre	R2X(cum)	R2Y(cum)	Q2(cum)
G1 vs. R1	PLS-DA	3	0.888	0.993	0.924
	OPLS-DA	1+1	0.776	0.964	0.788
G2 vs. R2	PLS-DA	3	0.942	0.999	0.977
	OPLS-DA	1+1	0.878	0.988	0.895
G1 vs. G2	PLS-DA	2	0.879	0.990	0.961
	OPLS-DA	1+1	0.879	0.990	0.953
R1 vs. R2	PLS-DA	3	0.962	1.000	0.999
	OPLS-DA	1+1	0.918	0.989	0.951

Note: pre: primary score; R2X(cum): interpretability for X-variable dataset; R2Y(cum): interpretability for Y-variable dataset; Q2: model predictability.

Table S4. Differential accumulation metabolites.

index	Compounds	Class II	Molecular Weight (Da)
Lmpp003662	Delphinidin-3-O-(6''-O-p-coumaroyl) glucoside	Anthocyanidins	611.140
Lmpp003815	Petunidin-3-O-(6''-O-p-Coumaroyl) glucoside	Anthocyanidins	625.155
pmb3041	Tricin-7-O-saccharic acid	Flavones	522.101
pmn001642	Kaempferol-3-O-(2''-O-acetyl) glucuronide	Flavonols	504.090
Zbsp004060	Apigenin-6-C-xyloside-8-C-arabinoside	Flavones	534.137
Lasp002993	Isobavachalcone glucoside	Chalcones	486.189
Smhp004476	Isovitexin-2''-O-xyloside*	Flavones	564.148
Lmnp102580	Apigenin-6-C-(2''-xylosyl) glucoside*	Flavones	564.148
Lmpp003789	Cyanidin-3-O-(6''-O-p-Coumaroyl) glucoside	Anthocyanidins	595.145
Lmtp002474	Apigenin-6-C-(2''-glucosyl) arabinoside	Flavones	564.148
mws1292	Isoschaftoside	Flavones	564.148
mws1434	Apigenin-6-C-glucoside (Isovitexin)*	Flavones	432.106

Zbsn004665	Apigenin-8-C-Glucoside (Vitexin)*	Flavones	432.106
ZBN0306	Quercetin-3-O-(4''-O-glucosyl) rhamnoside*	Flavonols	610.153
mws0059	Quercetin-3-O-rutinoside (Rutin)*	Flavonols	610.153
Lmgp004474	Genistein-7-O-galactoside-rhamnose	Isoflavones	578.164
pme0368	Apigenin-7-O-rutinoside (Isorhoifolin)	Flavones	578.164
pmp000413	Genistein-8-C-glucoside	Isoflavones	432.106
MWSslk146	4'-O-Glucosylvitexin	Flavones	594.159
Zbsp003407	Apigenin-6,8-di-C-glucoside (Vicenin-2)	Flavones	594.159
Lmlp002990	Isosaponarin(Isovitexin-4'-O-glucoside)	Flavones	594.159
Zmjp003291	Vitexin-2''-O-galactoside	Flavones	594.159
MWSHY0080	Luteolin-7-O-neohesperidoside (Lonicerin)*	Flavones	594.159
pmp000593	Luteolin-7-O-rutinoside*	Flavones	594.159
Lmsp004670	Kaempferol-3-O-glucoside-7-O-rhamnoside*	Flavonols	594.159
MWSHY0061	Kaempferol-3-O-neohesperidoside*	Flavonols	594.159
pme2960	Naringenin chalcone; 2',4,4',6'-Tetrahydroxychalcone	Chalcones	272.069
mws0914	3,5,7-Trihydroxyflavanone (Pinobanksin)	Flavanonols	272.069
HJAP120	Rhamnetin-3-O-Rutinoside*	Flavonols	624.169
MWSHY0064	Isorhamnetin-3-O-neohesperidoside*	Flavonols	624.169
Zblp004717	3'-methoxyquercetin-3-O-L-rhamnosyl (1→2)-glucopyranoside	Flavonols	624.169
Lmmp002463	Sexangularetin-3-O-glucoside-7-O-rhamnoside	Flavonols	624.169
Lmmp002963	6-C-Methylquercetin-3-O-rutinoside	Flavonols	624.169
pmn001583	Quercetin-3-O-robinobioside	Flavonols	610.153
pme1605	Kaempferol-3-O-robinobioside (Biorobin)*	Flavonols	594.159
MWSHY0017	Naringenin (5,7,4'-Trihydroxyflavanone) *	Flavanones	272.069
Zbsp007084	Butin; 7,3',4'-Trihydroxyflavanone*	Flavanones	272.069
Lmsn002815	Kaempferol-3-O-rutinoside (Nicotiflorin)*	Flavonols	594.159

Table S5. Metabolites identified by the flavonoid biosynthetic pathway.

index	Compounds	Class II	Ion mode	Molecular Weight
Zbsn004665	Apigenin-8-C-Glucoside	Flavones	[M-H]-	432.1056
mws1434	Apigenin-6-C-glucoside	Flavones	[M-H]-	432.1056
MWSHY0046	Quercetin-3-O-glucoside	Flavonols	[M+H]+	464.0955
mws0059	Quercetin-3-O-rutinoside	Flavonols	[M-H]-	610.1534
MWSHY0080	Luteolin-7-O-rhamnoside	Flavones	[M+H]+	594.1585
Lmsn002815	Kaempferol-3-O-rutinoside	Flavonols	[M-H]-	594.1585
Zmcp002839	Delphinidin-3,5,3'-Tri-O-glucoside	Anthocyanidins	[M]+	789.2084
Zbjp001957	Cyanidin-3,5-O-diglucoside	Anthocyanidins	[M]+	611.1607
Lmpp003789	Cyanidin-3-O-(6''-O-p-Coumaroyl) glucoside	Anthocyanidins	[M]+	595.1446
Lmpp003662	Delphinidin-3-O-(6''-O-p-coumaroyl) glucoside	Anthocyanidins	[M]+	611.1395
pme1201	Phloretin	Chalcones	[M-H]-	274.0841
pme2960	Naringenin chalcone	Chalcones	[M+H]+	272.0685
Zmpn002440	3,4,2',4',6'-Pentahydroxychalcone	Chalcones	[M-H]-	288.0634
Zbsp007084	Butin	Flavanones	[M+H]+	272.0685
MWSHY0017	Naringenin	Flavanones	[M+H]+	272.0685
mws0914	3,5,7-Trihydroxyflavanone	Flavanonols	[M-H]-	272.0685

Table S6. Statistical analysis of transcriptome sequencing quality in two periods of GDd and RDd.

Sample	Raw reads	Clean reads (%)	Q20(%)	Q30(%)	GC(%)
G1-1	37171614	37076564 (99.74%)	98.03%	93.93%	44.96%
G1-2	39792964	39682666 (99.72%)	98.05%	93.68%	44.67%
G1-3	42638104	42496950 (99.67%)	97.86%	93.27%	43.61%
G2-1	43112248	43038836 (99.83%)	96.82%	91.34%	45.37%
G2-2	47451156	47300688 (99.68%)	97.56%	93.26%	45.58%
G2-3	43649382	43525962 (99.72%)	97.75%	93.69%	45.50%
R1-1	49003992	48750178 (99.48%)	98.08%	93.92%	44.83%
R1-2	46927676	46782654 (99.69%)	97.92%	93.52%	45.37%
R1-3	36835456	36730894 (99.72%)	98.16%	94.29%	44.78%
R2-1	57266416	57030448 (99.59%)	97.69%	93.53%	45.90%
R2-2	45730252	45546094 (99.60%)	97.11%	92.28%	40.92%
R2-3	47974248	47816222 (99.67%)	97.37%	92.82%	45.34%

Table S7. Shared differential gene log2(fc) values.

ID	Symbol	G1vsR1 log2(fc)	G2vsR2 log2(fc)	G1vsG2 log2(fc)	R1vsR2 log2(fc)
Unigene0120143	--	-2.15	-1.98	-1.99	-1.82
Unigene0021771	--	-1.67	-1.90	-1.71	-1.93
Unigene0096130	CDC48C	1.75	4.63	-4.80	-1.93
Unigene0032996	UGT73E1	8.15	1.34	5.71	-1.11
Unigene0086395	AHL17	2.46	1.42	-1.52	-2.57
Unigene0025121	--	-1.92	-2.13	3.03	2.82
Unigene0111889	CAT6	2.08	1.80	1.96	1.68
Unigene0105383	--	-3.68	-4.95	-3.35	-4.62
Unigene0103370	PETF	-1.91	-1.51	1.16	1.57
Unigene0096501	--	-2.08	-4.72	-3.41	-6.05
Unigene0017530	--	15.06	1.70	11.85	-1.51
Unigene0087587	GDPDL7	-1.94	-1.33	-2.30	-1.69
Unigene0104565	MYOB5	-1.08	-1.67	-1.54	-2.13
Unigene0103918	--	-3.21	-11.31	-3.40	-11.50
Unigene0088946	--	-3.14	-10.81	-2.35	-10.02
Unigene0007188	psbB	7.15	1.68	8.08	2.61
Unigene0126171	ALMT10	2.37	5.49	-4.17	-1.06
Unigene0108088	--	-2.84	-9.83	-2.12	-9.11
Unigene0033295	PUP1	2.77	1.74	3.79	2.75
Unigene0085200	At1g67300	1.46	2.68	-3.24	-2.01
Unigene0089038	--	-4.57	6.70	-7.08	4.20
Unigene0085910	--	4.98	3.49	4.84	3.35
Unigene0118807	--	-3.52	-4.70	-3.37	-4.54
Unigene0091577	At2g36330	1.92	1.34	2.36	1.77
Unigene0020067	GALS1	-1.21	1.25	-1.02	1.43
Unigene0115882	ATL24	3.41	1.36	4.07	2.01
Unigene0105700	--	-1.56	-1.23	1.47	1.81
Unigene0011229	--	-3.83	-2.35	2.38	3.87
Unigene0122268	--	-1.22	-2.73	-1.48	-3.00
Unigene0096991	--	13.05	2.26	12.19	1.40
Unigene0085126	--	3.65	2.39	3.72	2.47
Unigene0119019	Pol	3.87	8.56	-7.59	-2.90
Unigene0090785	--	-5.41	-2.38	2.31	5.34
Unigene0090929	--	3.49	-2.76	3.45	-2.80

Unigene0013265	--	-1.85	-2.86	-2.00	-3.01
Unigene0014233	--	3.64	2.05	2.93	1.34
Unigene0011249	--	-7.72	9.99	-14.31	3.40
Unigene0095580	NUDT2	-1.52	-1.38	-1.33	-1.19
Unigene0057006	FLCY	-1.26	1.39	-1.45	1.20
Unigene0100306	CSA	2.58	3.42	-3.24	-2.40
Unigene0066617	Os01g0679700	1.32	1.18	1.18	1.05
Unigene0046387	OsI_28220	-1.18	-1.83	-1.30	-1.96
Unigene0107319	DCP5	11.48	-1.71	15.00	1.81
Unigene0060934	--	-1.59	-1.18	1.44	1.84
Unigene0008137	--	1.98	4.09	-3.67	-1.55
Unigene0126082	--	1.61	1.24	2.21	1.84
Unigene0103067	--	15.79	1.95	11.56	-2.27
Unigene0066654	ABCA7	1.62	1.76	-1.65	-1.51
Unigene0085375	SPA3	-1.87	-1.53	2.32	2.66
Unigene0004387	PIP2-5	2.41	1.93	3.16	2.68
Unigene0115294	RPA3	3.90	1.94	3.14	1.18
Unigene0039374	--	2.10	11.88	-13.17	-3.39
Unigene0112890	At1g56140	2.06	2.05	-1.72	-1.73
Unigene0019760	--	2.27	1.96	1.99	1.68
Unigene0104608	--	1.64	-1.26	4.45	1.55
Unigene0020550	--	1.66	2.61	-2.89	-1.94
Unigene0118296	HAL3	-1.14	-1.28	1.58	1.44
Unigene0093773	TY3B-I	5.55	3.81	-3.44	-5.18
Unigene0115992	Os03g0800700	-1.25	-1.10	1.94	2.08
Unigene0096600	--	3.95	5.19	-3.55	-2.30
Unigene0106768	--	2.85	2.05	1.96	1.16
Unigene0061082	G9	-5.28	12.03	-14.22	3.09
Unigene0120060	PRXIIC	8.31	3.40	7.06	2.15
Unigene0002817	Os03g0210200	3.66	2.27	3.00	1.61
Unigene0083851	PCK1	1.91	2.07	1.95	2.11
Unigene0082146	--	4.26	-1.79	4.24	-1.82
Unigene0098088	UBC24	1.45	-1.15	1.37	-1.22
Unigene0125698	LECRK4	4.11	-2.40	3.90	-2.61
Unigene0086648	PDIL5-1	2.12	2.06	1.69	1.63
Unigene0092321	Srrm2	-1.42	-1.34	-1.67	-1.59
Unigene0011996	PCR2	-3.54	-2.49	1.14	2.18
Unigene0098647	PDR17	-1.39	2.20	-1.88	1.71
Unigene0119006	--	13.93	3.88	11.86	1.81
Unigene0089018	NTMC2T6.1	-1.66	-1.83	-1.11	-1.28
Unigene0094857	RGA1	3.86	6.69	-4.15	-1.33
Unigene0075691	--	-2.09	13.72	-13.70	2.11
Unigene0095987	--	1.07	12.68	-13.52	-1.91
Unigene0104829	--	-2.75	-2.68	2.26	2.33
Unigene0026252	--	1.81	13.87	-14.32	-2.26
Unigene0101831	NPF3.1	2.21	2.07	3.10	2.96
Unigene0124769	AAMT2	6.01	-3.35	5.02	-4.35
Unigene0084898	PUMP5	-2.75	-2.42	4.16	4.49
Unigene0099556	AVT6C	1.31	2.19	-2.19	-1.31
Unigene0019535	--	-3.15	-3.15	2.92	2.91
Unigene0020206	TIP2-1	-2.21	3.91	-4.10	2.02
Unigene0091326	--	-1.97	3.13	1.61	6.71
Unigene0107766	--	4.60	3.95	4.15	3.50

Unigene0092190	--	-2.22	-5.41	2.18	-1.00

Table S8. Expression of structural genes related to flavonoid biosynthesis pathway.

Gene	Gene-id	Gene	Gene-id	Gene	Gene-id
PAL	Unigene0026391	*CYP75B3*	Unigene0014633	*ANS*	Unigene0050010
PAL	Unigene0026392	*CYP75B3*	Unigene0124953	*ANR*	Unigene0008594
4CL1	Unigene0013889	*CYP75B137*	Unigene0082641	*FLS*	Unigene0024995
4CL1	Unigene0115617	*CYP75B137*	Unigene0083940	*THT*	Unigene0007904
4CL1	Unigene0013887	*CYP75A1*	Unigene0021026	*UGT77B2*	Unigene0086860
4CL1	Unigene0020313	*CHI*	Unigene0088693	*3GT*	Unigene0123416
4CL2	Unigene0102276	*CHI*	Unigene0088694	*5GT*	Unigene0007515
CHS	Unigene0009970	*CHI3*	Unigene0119545	*5GT*	Unigene0007582
CHS	Unigene0009971	*F3H-2*	Unigene0010253	*5GT*	Unigene0056697
CHS	Unigene0033966	*F3H-2*	Unigene0071875	*5GT*	Unigene0120074
CHS2	Unigene0084168	*DFR1*	Unigene0052001	*3AT1*	Unigene0021833
CHS2	Unigene0018266	*DFR2*	Unigene0101703	*3AT1*	Unigene0110169
CHS8	Unigene0021168	*DFR3*	Unigene0122364	*3AT2*	Unigene0110547
CHS-DII	Unigene0023618	*CCOAOMT1*	Unigene0006100	*GT1*	Unigene0055257
CYP73A16	Unigene0102753	*CCOAOMT1*	Unigene0103871	*FG2*	Unigene0093792
CYP98A1	Unigene0117955	*CCOAOMT*	Unigene0113495		

Table S9. Transcription factors of different TFs families.

TFs_fanmily	Gene_ID	TFS	TFs_fanmily	Gene_ID	TFS
GRAS	Unigene0113433	GRAS1	MYB	Unigene0092013	MYB1
	Unigene0015469	GRAS2		Unigene0003420	MYB2
	Unigene0022175	GRAS3	NF-YB	Unigene0024190	NF-YB1
	Unigene0075427	GRAS4		Unigene0088503	NF-YB2
	Unigene0124131	GRAS5		Unigene0000698	NF-YB3
	Unigene0087632	GRAS6		Unigene0030551	NF-YB4
	Unigene0097734	GRAS7		Unigene0085622	NF-YB5
	Unigene0007558	GRAS8	WRKY	Unigene0118827	WRKY1
bHLH	Unigene0089414	bHLH1		Unigene0099858	WRKY2
	Unigene0089036	bHLH2	bZIP	Unigene0091293	Bzip1
	Unigene0093288	bHLH3		Unigene0122331	bZIP2
	Unigene0100630	bHLH4		Unigene0123754	bZIP3
	Unigene0108271	bHLH5		Unigene0007474	bZIP4
SAP	Unigene0087176	SAP1		Unigene0117141	bZIP5
	Unigene0002820	SAP2		Unigene0123957	bZIP6
	Unigene0011943	SAP3	FAR1	Unigene0086180	FAR11
	Unigene0036481	SAP4		Unigene0022711	FAR12
	Unigene0097594	SAP5	NF-YC	Unigene0094295	NF-YC1
	Unigene0109705	SAP6		Unigene0111409	NF-YC2
	Unigene0124552	SAP7		Unigene0094293	NF-YC3
	Unigene0086627	SAP8		Unigene0110937	NF-YC4
	Unigene0096158	SAP9		Unigene0115212	NF-YC5
ARF	Unigene0059009	ARF1		Unigene0121082	NF-YC6
	Unigene0038515	ARF2	G2-like	Unigene0114265	G2-like1
	Unigene0123044	ARF3		Unigene0105111	G2-like2
	Unigene0116846	ARF4		Unigene0123319	G2-like3
	Unigene0059401	ARF5		Unigene0105813	G2-like4
ERF	Unigene0083614	ERF1	NAC	Unigene0009921	NAC
	Unigene0011366	ERF2	DBB	Unigene0125739	DBB1

	Unigene0032998	ERF3		Unigene0082742	DBB2
	Unigene0027234	ERF4	GeBP	Unigene0015550	GeBP1
	Unigene0081584	ERF5		Unigene0010690	GeBP2
	Unigene0089603	ERF6	LBD	Unigene0105997	LBD
	Unigene0039577	ERF7	NF-YA	Unigene0052929	NF-YA1
	Unigene0091000	MYB_related1		Unigene0098140	NF-YA2
	Unigene0010835	MYB_related2		Unigene0015721	Trihelix1
MYB_related	Unigene0107327	MYB_related3	Trihelix	Unigene0103068	Trihelix2
	Unigene0033245	MYB_related4		Unigene0050387	Trihelix3
	Unigene0119366	MYB_related5	CO-like	Unigene0014550	CO-like
	Unigene0081778	MYB_related6	EIL	Unigene0118380	EIL
B3	Unigene0091262	B3	SBP	Unigene0096605	SBP

Table S10. Screening of key genes for flavonoid biosynthesis in *D. devonianum*.

Gene	Gene-id	Ko ID（EC）	Name
CHS2	Unigene0084168	Ko0660[EC:2.3.1.74]	Chalcone synthase
CHI	Unigene0088694	Ko1859[EC:5.5.1.6]	Chalcone isomerase
4CL	Unigene0115617	Ko1904[EC:6.2.1.12]	4-coumarate--CoA ligase
GT1	Unigene0055257	K12938[EC:2.4.1.-]	Anthocyanidin 5,3-O-glucosyltransferase
CHS8	Unigene0021168	Ko0660[EC:2.3.1.74]	Chalcone synthase
CHS	Unigene0033966	Ko0660[EC:2.3.1.74]	Chalcone synthase
UGT77B2	Unigene0086860	K12930[EC:2.4.1.115]	Anthocyanidin 3-O-glucosyltransferase
CHI3	Unigene0119545	Ko1859[EC:5.5.1.6]	Chalcone isomerase

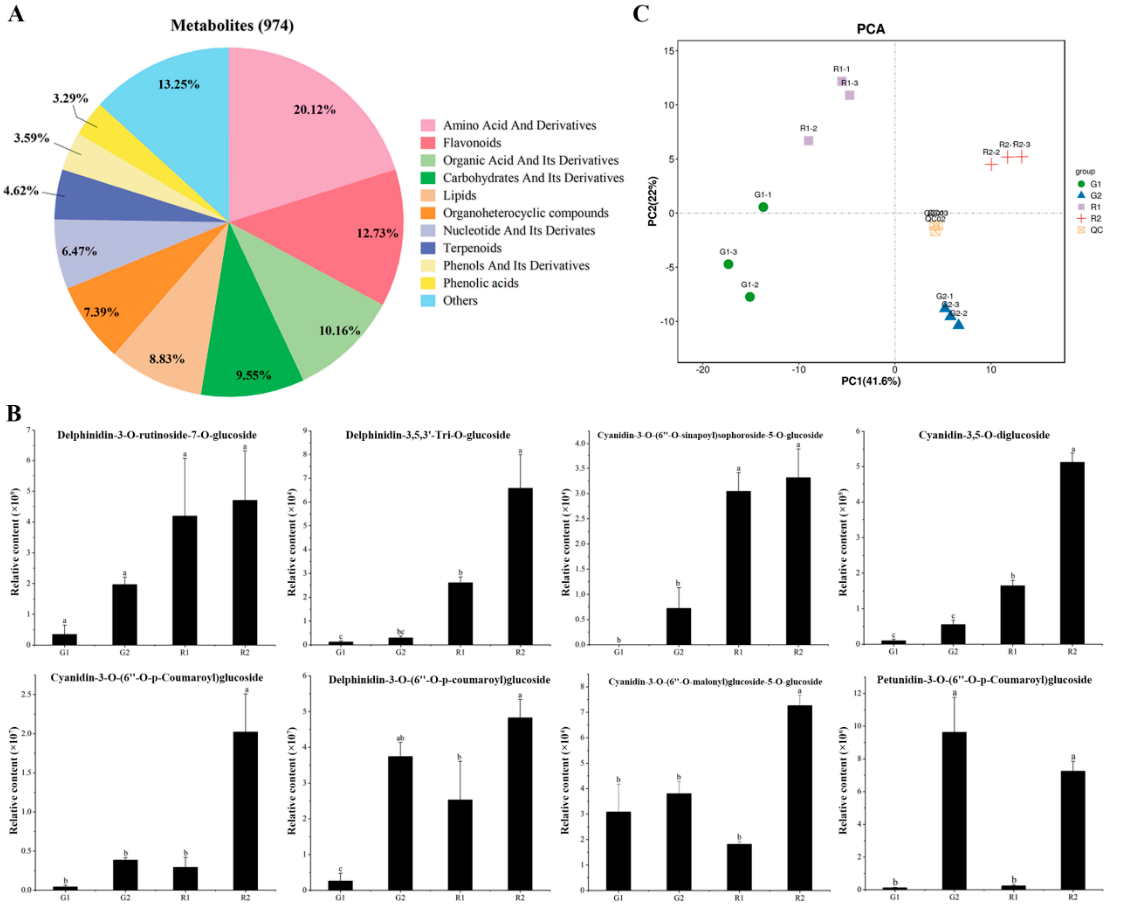

Figure S1. (A) Classification statistics of identified broad-targeted metabolites. **(B)** Relative content of anthocyanidins in different samples. **(C)** PCA Analysis.

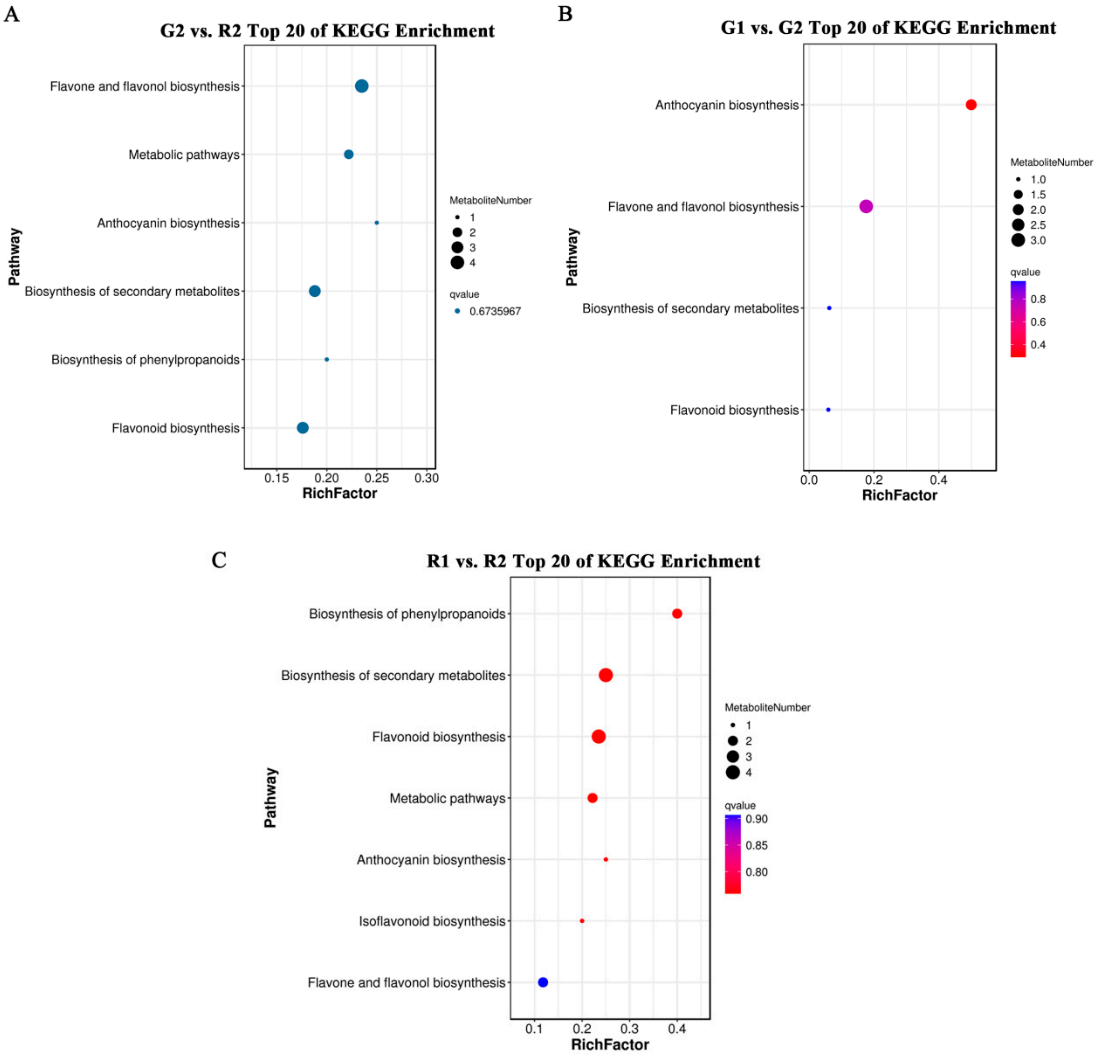

Figure S2. KEGG enrichment pathway of DAFs. **(A)** KEGG enrichment pathway of DAFs in G2 vs. R2. **(B)** KEGG enrichment pathway of DAFs in G1 vs. G2. **(C)** KEGG enrichment pathway of DAFs in R1 vs. R2.

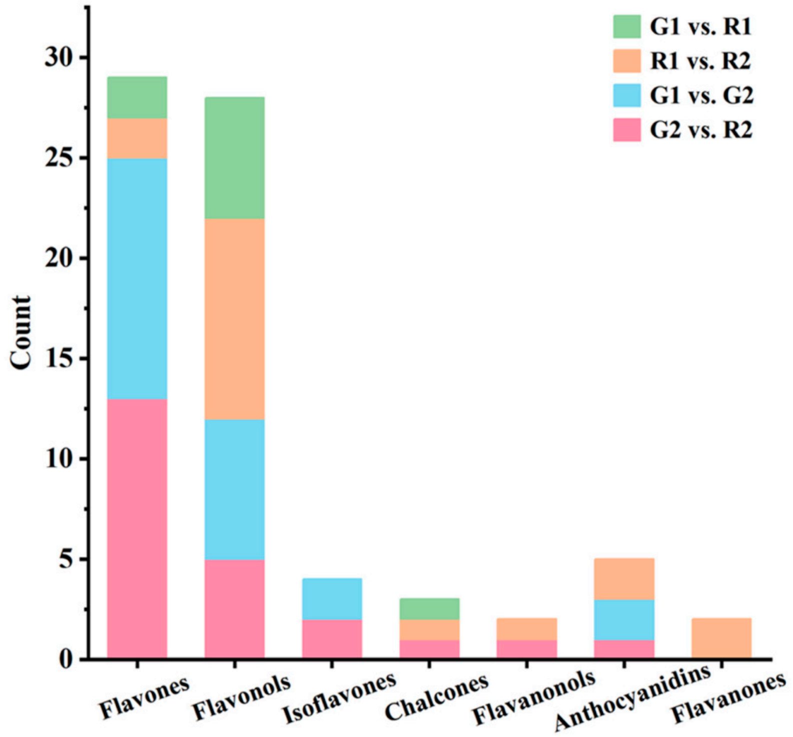

Figure S3. Differential Metabolite Statistical Analysis Chart.

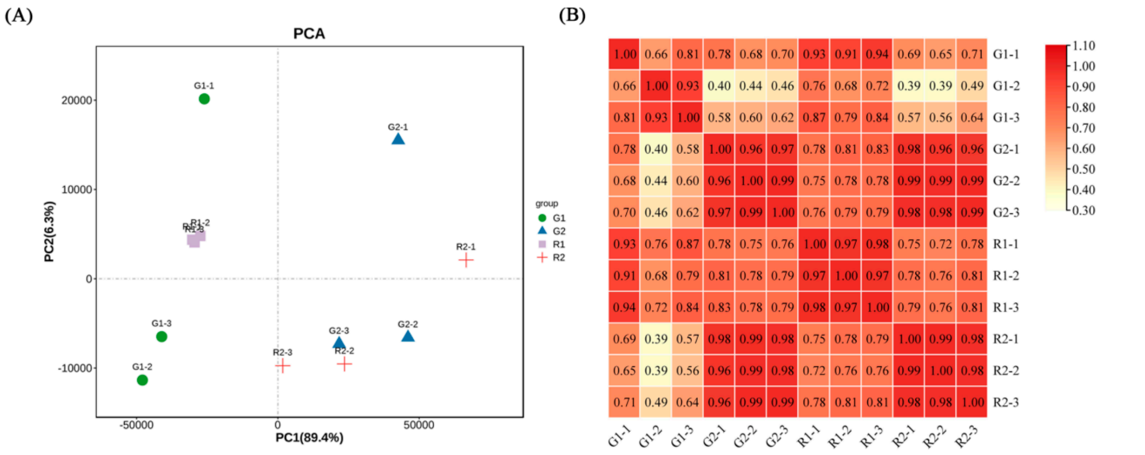

Figure S4. Correlation analysis of transcriptome data. **(A)** PCA analysis. **(B)** Pearson correlation analysis between all samples.

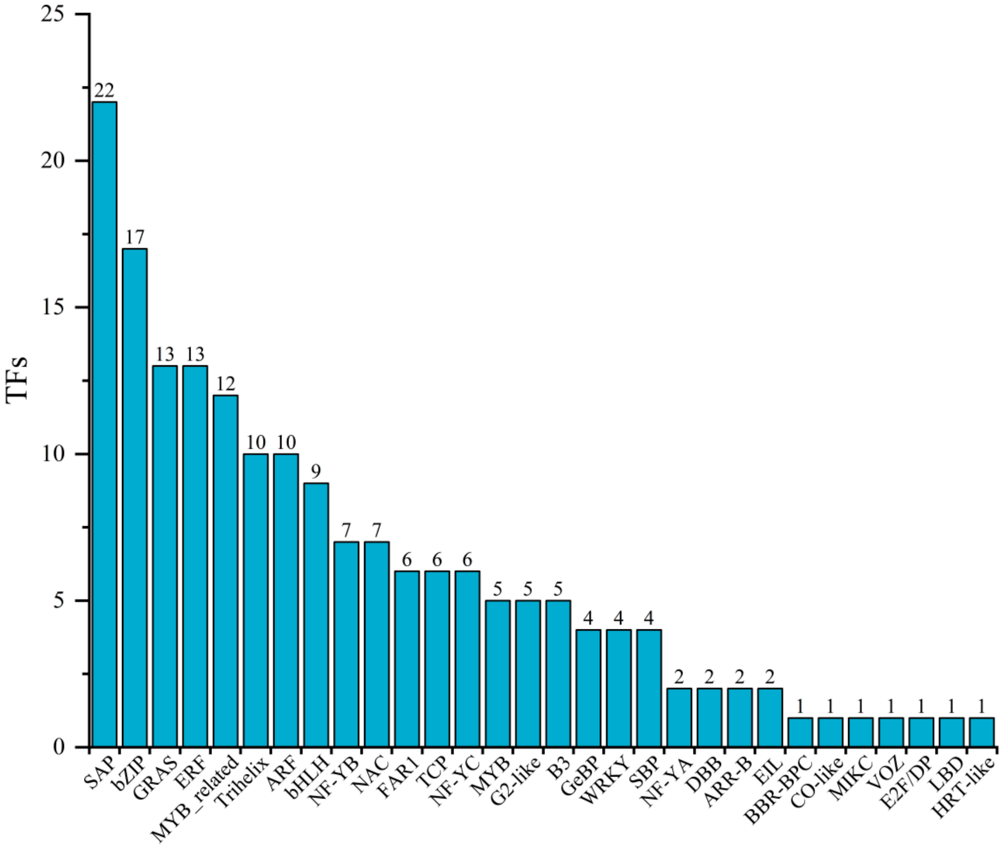

Figure S5. Statistics of the number of gene families of TFs.
